# The 9-1-1 checkpoint clamp coordinates resection at DNA double strand breaks

**DOI:** 10.1093/nar/gkv409

**Published:** 2015-04-29

**Authors:** Greg H.P. Ngo, David Lydall

**Affiliations:** Institute for Cell and Molecular Biosciences (ICaMB), Medical School, Newcastle University, Newcastle upon Tyne, NE2 4HH, UK

## Abstract

DNA-end resection, the generation of single-stranded DNA at DNA double strand break (DSB) ends, is critical for controlling the many cellular responses to breaks. Here we show that the conserved DNA damage checkpoint sliding clamp (the 9-1-1 complex) plays two opposing roles coordinating DSB resection in budding yeast. We show that the major effect of 9-1-1 is to inhibit resection by promoting the recruitment of Rad9^53BP1^ near DSBs. However, 9-1-1 also stimulates resection by Exo1- and Dna2-Sgs1-dependent nuclease/helicase activities, and this can be observed in the absence of Rad9^53BP1^. Our new data resolve the controversy in the literature about the effect of the 9-1-1 complex on DSB resection. Interestingly, the inhibitory role of 9-1-1 on resection is not observed near uncapped telomeres because less Rad9^53BP1^ is recruited near uncapped telomeres. Thus, 9-1-1 both stimulates and inhibits resection and the effects of 9-1-1 are modulated by different regions of the genome. Our experiments illustrate the central role of the 9-1-1 checkpoint sliding clamp in the DNA damage response network that coordinates the response to broken DNA ends. Our results have implications in all eukaryotic cells.

## INTRODUCTION

Double strand breaks (DSBs) are one of the most toxic forms of DNA damage because both strands of DNA are damaged and therefore there is no undamaged strand of DNA available to act as a template for repair. Spontaneous or environmentally induced DSBs may cause cell death, genetic instability and cancer. On the other hand, programmed DSBs stimulate meiotic recombination and the genetic rearrangements important for immune system maturation in mammals or mating type switches in yeasts. It is therefore critical to understand how the cellular response to DSBs is coordinated in different contexts.

The eukaryotic DNA damage response (DDR) network coordinates responses to damaged DNA by regulating DNA repair, checkpoint-dependent cell cycle arrest and transcription (1,2). At DSBs, in yeast/mammalian cells the DDR initiates when the MRX/MRN (Mre11-Rad50-Xrs2/MRE11-RAD50-NBS1) complex binds to ‘blunt’ DSBs and activates the transducer checkpoint kinase Tel1/ATM. MRX also controls nuclease activities that help generate single-stranded DNA (ssDNA), another stimulus for DNA damage checkpoint activation.

The generation of ssDNA at DSBs by the process of resection is critically important for determining cellular responses to DSBs (3). In particular, resection affects DNA repair pathway choice by either non-homologous end joining (NHEJ, which requires limited or no resection) or homology-directed repair (HDR, which requires extensive resection) (4–7). Resection also controls the switch between blunt dsDNA-end-activated Tel1/ATM signalling and ssDNA-activated Mec1-Ddc2/ATR-ATRIP signalling (8–10).

Resection at DSBs is initiated by MRX^MRN^-Sae2^CtIP^, whereas more extensive DNA resection requires the nuclease Exo1 and/or the nuclease/helicase pair of Dna2 and Sgs1^BLM^ (3,11). It is critical that Exo1 and Dna2-Sgs1 resection activities are properly regulated because excess levels of ssDNA are potentially very harmful to cell survival by inducing cell death and mutation (12–17).

The conserved, proliferating cell nuclear antigen related 9-1-1 checkpoint sliding clamp is composed of three subunits Ddc1/RAD9, Mec3/HUS1 and Rad17/RAD1. The clamp is loaded onto DNA by the Rad24-RFC (RAD17-RFC) clamp loader complex and is one of the earliest sensors recruited to sites of DNA damage (18,19). Once loaded onto the DNA, the 9-1-1 clamp performs at least three functions to activate checkpoint kinase cascades. First, 9-1-1 activates Mec1 directly via the unstructured C terminal domain of Ddc1. Second, 9-1-1 recruits Dpb11/TopBP1 near DNA lesions via phosphorylated Ddc1 (20). Third, the 9-1-1 clamp and the clamp loader Rad24-RFC promote resection (21–23).

**Figure 1.**
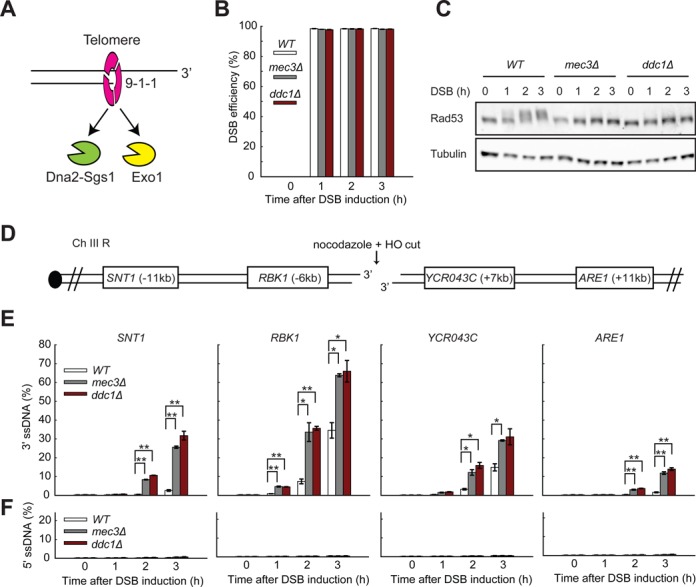
The 9-1-1 complex inhibits resection of DSBs. (**A**) The 9-1-1 complex promotes resection at uncapped telomeres by stimulating Exo1 and Dna2-Sgs1 recruitment to ssDNA (model based on Ngo *et al*. (24)). (**B**) The efficiencies of DSB induction at the *MAT* locus. (**C**) Yeast strains of the indicated genotypes were subjected to western blot analysis with anti-Rad53 and anti-tubulin antibodies following DSB induction. (**D**) Map of chromosome III showing an HO endonuclease site and loci examined in this study. (**E,F**) Analysis of 3′ ssDNA and 5′ ssDNA accumulation in JKM179 strains (see strain list in Supplementary Tables S1 and S2) at the indicated loci. All DSB experiments were performed in nocodazole arrested cells. The data plotted are the means and the range from two strains. *P*-values were calculated using two-tailed unpaired T test. *P < 0.05, **P < 0.01.

Recently, we showed that 9-1-1 promotes resection by stimulating both Dna2-Sgs1- and Exo1-dependent resection near uncapped telomeres in yeast cells, *in vivo*, and using human proteins *in vitro* (Figure 1A) (24). We suggested that the binding of the 9-1-1 complex near sites of resection served to initiate a positive feedback loop to generate ssDNA for DNA damage checkpoint maintenance. Importantly, we showed that the human 9-1-1 complex stimulated the activities of DNA2 and EXO1 on DNA substrates *in vitro* (24), showing that the effect of 9-1-1 is direct and conserved. However, the effects of the 9-1-1 complex on resection *in vivo*, in budding yeast, have nearly all been shown using telomere-defective, *cdc13-1* mutants. Whether 9-1-1 affects resection at DSBs *in vivo* remained unclear. One study reported that Rad17 had no role in DSB resection (25), whereas another found that Rad24 stimulated resection (26).

To clarify how the 9-1-1 complex affects resection at DSBs, *in vivo*, we have now analysed resection at DSBs in non-dividing yeast cells. Examination of non-dividing, nocodazole-arrested cells ensured that any effects of 9-1-1 were direct rather than indirectly caused by differences in cell cycle position between strains. Our experiments, in which we inactivated as many as four independent components of the DDR network, show that the 9-1-1 complex plays a complex role at DSBs. Our major finding is that 9-1-1 inhibits DSB resection by promoting the recruitment of Rad9^53BP1^ near DSBs. In addition, in the absence of Rad9, we found that 9-1-1 stimulates DSB resection by both Dna2-Sgs1 and Exo1, as at telomeres. The role of 9-1-1 inhibiting resection is not observed near uncapped telomeres because less Rad9^53BP1^ is recruited near uncapped telomeres in comparison to near DSBs. Our data illustrate the central role played by the conserved 9-1-1 complex in the network coordinating DNA resection and checkpoint activation at DNA ends.

## MATERIALS AND METHODS

### Yeast strains and plasmids

All experiments were performed on *Saccharomyces cerevisiae* JKM179 or W303 background strains (Supplementary Tables S1 and S2).

### DNA damage induction and quantitative amplification of single-stranded DNA

For DSB studies, yeasts were first arrested in Yeast Extract Peptone (YEP) 3% lactate (supplemented with adenine) with 15 μg/ml nocodazole for 3 h at 30°C before addition of galactose (to a final concentration of 2%) to induce HO-endonuclease. We determined that few cells escaped nocodazole arrest by counting 4′, 6-diamidino-2-phenylindole stained cells. For telomere uncapping studies, *cdc13-1* strains in Yeast Extract Peptone Dextrose (YEPD) were arrested in G1 with alpha factor at 23°C and released into 36°C. Quantitative Amplification Of Single-stranded DNA (QAOS) analyses at uncapped telomeres and DSBs were carried out as previously described (27–29), and in the DSB studies the ssDNA amounts were normalized to DSB efficiencies as previously described (30). The primers used for QAOS at DSBs are listed in Supplementary Tables S3 and S4. The efficiencies of DSB induction at the *MAT* and *URA3* loci were determined using primer pairs which span the HO recognition sites (Supplementary Table S4), normalized to amount of DNA at *PAC2*, a control locus.

### Chromatin immunoprecipitation

Chromatin immunoprecipitation was performed as described (31). Briefly, yeast cultures were treated with formaldehyde (1% final) for 15 min before glycine (0.32% final) was added. The cells were washed twice in Tris-buffered saline (TBS) buffer, once in FA lysis buffer (pH 7.5, 50 mM HEPES, 150 mM NaCl, 1 mM EDTA, 1% (v/v) Triton X-100, 0.1% (w/v) sodium deoxycholate and 0.1% (w/v) sodium dodecylsulphate [SDS]), spun down and frozen at −80°C. Cell pellets were lysed in FA lysis buffer (with 2 mM phenylmethylsulfonyl fluoride, PMSF) by bead beating using a Precellys 24 homogenizer (Precellys). The cell lysates were incubated with anti-HA (ab9110, Abcam) or anti-Myc (ab32, Abcam) antibodies together with protein G Dynabeads (Invitrogen) on a wheel at 4°C overnight. The immunoprecipitates were washed five times in FA lysis buffer and eluted in ChIP elution buffer (50 mM Tris·Cl, pH 7.5, 10 mM EDTA and 1% (w/v) SDS). The samples were treated with proteinase K and incubated overnight at 62°C to reverse the crosslinks. Immunoprecipitated DNA was purified using PCR purification kit (QIAGEN) and quantified using the SYBR Green qPCR SuperMIX-UDG w/ROX kit (11744500, Invitrogen) by a SteponePlus qPCR machine (Life Technologies; all samples were also normalized by input DNA, quantified on the same plates).

### Western Blots

Protein extracts were prepared by trichloroacetic acid (TCA) precipitation. Briefly, cells were resuspended in 10% TCA and mechanically broken using glass beads. Protein suspensions in Laemmli buffer were boiled for 3 min, spun down for 10 min and the supernatant were loaded onto 7.5% Mini-PROTEAN TGX Gels (Bio-Rad). The proteins were transferred to Hybond-ECL membranes (GE Healthcare) and probed with anti-Rad53 (sc-6749, Santa Cruz) and anti-tubulin antibodies (from Keith Gull, Oxford University).

## RESULTS

### The 9-1-1 complex inhibits resection at DSBs

We have shown that the 9-1-1 complex stimulates Exo1- and Dna2-Sgs1-dependent resection *in vivo* and *in vitro* (Figure 1A) (24). To extend these studies to DSBs *in vivo*, we deleted *MEC3* and *DDC1* (encoding two subunits of the 9-1-1 complex) in the widely used JKM179 genetic background (25). In the JKM179 strain background, galactose-inducible expression of the HO endonuclease leads to a DSB being induced at the *MAT* locus of chromosome III. Additionally, the JKM179 strain lacks *HML* and *HMR*, the silent mating type donor loci, and therefore cannot repair the DSB by gene conversion. *9-1-1* mutants are checkpoint deficient (32), and therefore we performed all the experiments in the presence of the spindle poison nocodazole to arrest cells in M phase and to ensure that any differences in ssDNA measurement were not affected by different cell cycle positions in different strains (33,34).

We arrested wild-type, *mec3Δ* and *ddc1Δ* strains for 3 h in nocodazole before adding galactose to induce HO expression. We found that DSBs were induced with high efficiencies, reaching over 95% in all strains 1 h after addition of galactose (Figure 1B). DSB induction results in the phosphorylation and activation of the downstream checkpoint effector kinase Rad53 (35). As expected, 2 h following DSB induction, we observed strong DNA damage checkpoint activation in the wild-type strain, as shown by a mobility shift of Rad53 (Figure 1C). Consistent with an essential role of 9-1-1 in activating the DSB-induced checkpoint, we observed no Rad53 phosphorylation in *mec3Δ* or *ddc1Δ* mutants (Figure 1C) (25,36). We conclude that, as expected, the 9-1-1 complex does not affect HO-induced DSB formation and is required for DSB-induced DNA damage checkpoint activation.

To examine resection, we measured ssDNA accumulation at loci on both sides of the break using QAOS (37) (Figure 1D and E, and Supplementary Figure S1A). In wild-type cells, we observed the accumulation of detectable levels of 3′ ssDNA, 6kb and 7kb from the break sites at *RBK1* and *YCR043C*, by 2 h after DSB induction (Figure 1E). As expected, more distal to the break, 11kb away, at *SNT1* and *ARE1* ssDNA was detected later in the wild-type (WT) strains, by 3 h (Figure 1E). Our data show that the rate of DSB resection in the wild-type strains is about 3.5 kb/h (11 kb/3 h at *SNT1*/*ARE1*, 6–7 kb/2 h at *RBK1*/*YCR043C* and 9 kb/3 h at *ELO2*), consistent with previous findings (38). As expected, we did not observe 5′ ssDNA at any locus in all the strains (Figure 1F). Our results also show that even though the DSB efficiency is almost 100%, the level of 3′ ssDNA only reached 15–35% in the WT strains after 3 h at *YCR043C* and *RBK1*, consistent with previous studies (34,39,40). This finding suggests that only a fraction of breaks undergo resection or that not all breaks are resected at fast rates. Intriguingly, we also noticed an asymmetry in resection at either side of the break, with 3′ ssDNA generation being more efficient on the left than on the right side of the break (Figure 1E). This phenomenon has been observed at the *MAT* locus with Rad51 binding (a marker for ssDNA) more efficiently to the left side of the break in some experiments (41), but the mechanism underlying this asymmetry remains unclear.

Interestingly, and in contrast to our observations at uncapped telomeres, the 9-1-1 complex inhibited, rather than stimulated resection at DSBs (Figure 1E versus Supplementary Figure S1B) (21,23,24). For example at 3 h, at *SNT1*, about 11 kb from the DSB, less than 3% ssDNA was observed in WT cells but over 20% was observed in *mec3Δ* and *ddc1Δ* mutants (Figure 1E). By T test, this was statistically significant (Figure 1E). In addition, the statistically significant difference we detected between WT and *9-1-1* mutants, at the *SNT1* locus at 3 h, is replicated at the *SNT1* locus at 2 h, and at *RBK1, YCR043C* and *ARE1* at all time points when ssDNA is present. In contrast to at DSBs, near uncapped telomeres*, mec3Δ* mutants generally showed less than half the ssDNA observed in WT cells at all loci and times (Supplementary Figure S1B). Interestingly, the rate of telomere resection near telomeres in wild-type strains is about 8 kb/h (30 kb/4 h, see *DUG1*) about twice as fast as at DSBs. This suggests that the telomere environment is more permissive for resection than other chromosomal locations.

In telomere uncapping experiments, checkpoint-deficient *mec3Δ* cells were held in late mitosis by *cdc15-2*-induced arrest (Supplementary Figure S1B), whereas in DSB experiments (Figure 1), *mec3Δ* cells were arrested earlier in mitosis by nocodazole. Therefore, one trivial reason to explain the contradictory conclusions drawn about the role of 9-1-1 on resection was that the effects of 9-1-1 were affected by cell cycle position (*cdc15-2* versus nocodazole). To address this issue we chose to uncap telomeres of *cdc13-1* cells in nocodazole-arrested cells. We performed these experiments at low temperatures of 32 and 34°C because nocodazole does not maintain arrest very well at higher temperatures (42) and because *cdc13-1* still induces efficient uncapping at these lower temperatures (24). We observed less telomeric ssDNA generation in nocodazole-arrested cells in comparison with *cdc15-2*-arrested cells (Supplementary Figure S2 versus Supplementary Figure S1B). The low levels of ssDNA in *cdc13-1* mutants in nocodazole has been reported before and is likely due to lower level of telomere uncapping in cells that have not gone through S phase (43). Nevertheless, in nocodazole, *cdc13-1 mec3Δ* cells still accumulated less ssDNA than *cdc13-1* cells (Supplementary Figure S2). This contrasts to what is observed at DSBs in nocodazole where *mec3Δ* cells accumulate higher levels of ssDNA. We conclude that at uncapped telomeres 9-1-1 stimulates extensive resection (21,23,24) whereas at DSBs the 9-1-1 complex inhibits DNA resection.

### The 9-1-1 complex inhibits Dna2-Sgs1 but stimulates Exo1 at DSBs

**Figure 2.**
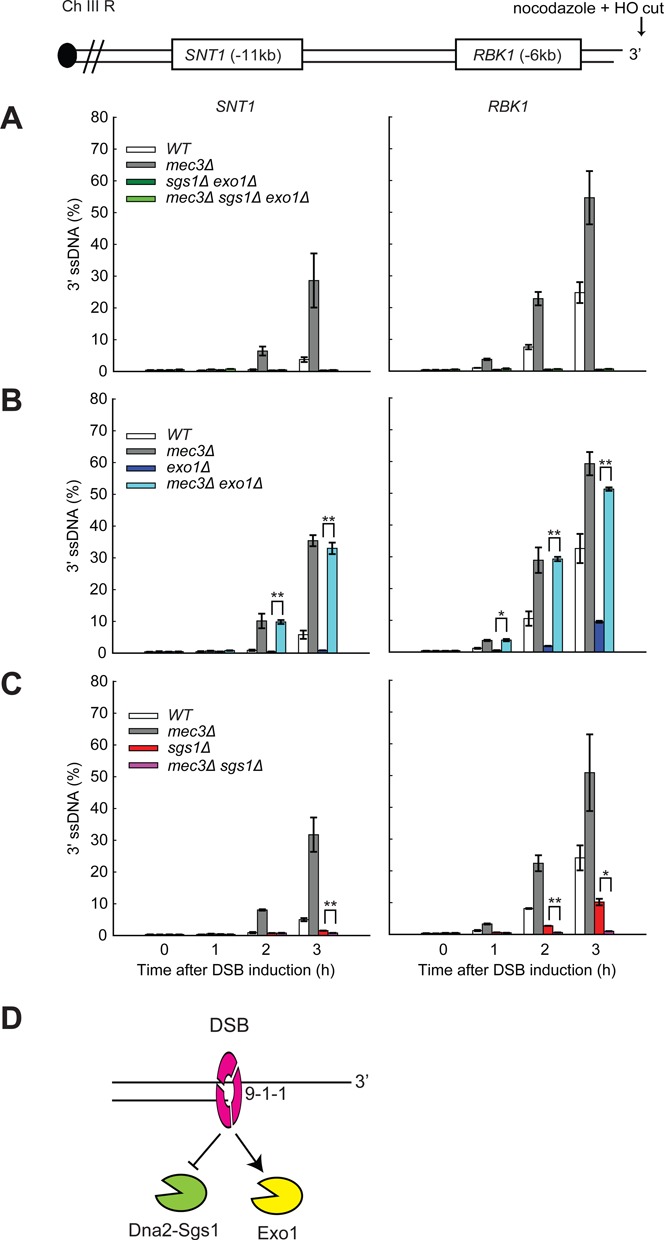
The 9-1-1 complex inhibits Dna2-Sgs1 but stimulates Exo1 at DSBs. (**A-C**) Analysis of 3′ ssDNA accumulation in JKM179 strains at the indicated loci. The data plotted and the *P*-values are as described in Figure 1. (**D**) The 9-1-1 complex inhibits Dna2-Sgs1 but stimulates Exo1 at DSBs.

To begin to understand how 9-1-1 inhibits DSB resection, we examined the interplay between 9-1-1 and Dna2-Sgs1 or Exo1, the two major nuclease activities involved in extensive resection. We deleted *MEC3* in *exo1Δ, sgs1Δ* or *sgs1Δ exo1Δ* mutant backgrounds and compared ssDNA levels in single, double and triple mutants (Figure 2A–C). In agreement with published results (5,9,38), we found that extensive DSB resection is totally dependent on Dna2-Sgs1 and Exo1 (compare WT with *sgs1Δ exo1Δ* strains, Figure 2A). Importantly the *mec3Δ* mutation did not permit nucleases other than Dna2-Sgs1 and Exo1 to generate ssDNA since *mec3Δ sgs1Δ exo1Δ* and *sgs1Δ exo1Δ* strains each generated similarly low amounts of ssDNA (Figure 2A).

Interestingly, we observed differing effects of Mec3 on Exo1- or Dna2-Sgs1-dependent resection. Figure 2B shows that Exo1 is critical for generating ssDNA 6 kb and 11 kb from the DSB, with much lower levels of ssDNA being detected in *exo1Δ* versus WT strains. We found that *mec3Δ* mutation strongly increased resection in *exo1Δ* strains (compare *mec3Δ exo1Δ* with *exo1Δ* strains). Since in *exo1Δ* strains resection is totally dependent on Dna2-Sgs1, we can conclude that the major inhibitory effect of Mec3 on resection is to inhibit Dna2-Sgs1-dependent nuclease activity.

We also examined how *mec3Δ* affects resection in *sgs1Δ* strains, where all the resection is due to Exo1. *sgs1Δ*, like *exo1Δ*, strongly reduced ssDNA in comparison with WT cells (compare the effects of Sgs1 and Exo1 at the *RBK1* locus, in Figure 2C and B). The comparison allows us to conclude that Exo1 and Dna2-Sgs1 make similar contributions to resection in WT (*MEC3*) strains. Remarkably, however, 9-1-1 had opposite effects in *sgs1Δ* versus *exo1Δ* strains. *mec3Δ* largely eliminated ssDNA accumulation in *sgs1Δ* strains (compare *mec3Δ sgs1Δ* with *sgs1Δ* strains, Figure 2C), in stark contrast to the effect in *exo1Δ* strains where *mec3Δ* enhanced ssDNA accumulation (Figure 2B). To confirm the effects of Mec3 in the absence of Dna2/Sgs1, we also examined the effect of Mec3 in *dna2Δ* (*pif1Δ* background) strains (Supplementary Figure S3A), as *dna2Δ* and *sgs1Δ* mutations show similar effects on DSB resection (38). Importantly, *mec3Δ* largely eliminated ssDNA accumulation in *dna2Δ* strains (compare *mec3Δ dna2Δ* with *dna2Δ* strains, Supplementary Figure S3A). Since in *sgs1Δ* (and *dna2Δ*) strains resection is totally dependent on Exo1, we conclude that the 9-1-1 complex stimulates Exo1-dependent resection at DSBs.

Overall, examination of DSB resection in *exo1Δ* or *sgs1Δ*/*dna2Δ* mutants shows that 9-1-1 plays a positive role on Exo1-dependent resection but a negative role on Dna2-Sgs1-dependent resection (Figure 2D). We suggest that at DSBs the role of 9-1-1 in inhibiting Dna2-Sgs1-dependent resection predominates over its role in stimulating Exo1 and that this explains why 9-1-1 inactivation leads to increased DSB resection. In contrast at uncapped telomeres, 9-1-1 contributes more to stimulating resection than inhibiting resection (21,23,24).

### The 9-1-1 complex recruits more Rad9^53BP1^ near DSBs than uncapped telomeres

It was unclear why the effects of 9-1-1 on resection were so dramatically different at DSBs versus uncapped telomeres. One of the functions of 9-1-1 is to activate the central transducer kinase Mec1 (20). A recent study suggests that active Mec1 inhibits resection by promoting the recruitment of Rad9^53BP1^ to DSBs (34). The clamp loader Rad24^RAD17^ also stimulates Rad9^53BP1^ binding to DSBs (44). We therefore hypothesized that 9-1-1 might, like Mec1, inhibit DSB resection by promoting Rad9^53BP1^ recruitment.

**Figure 3.**
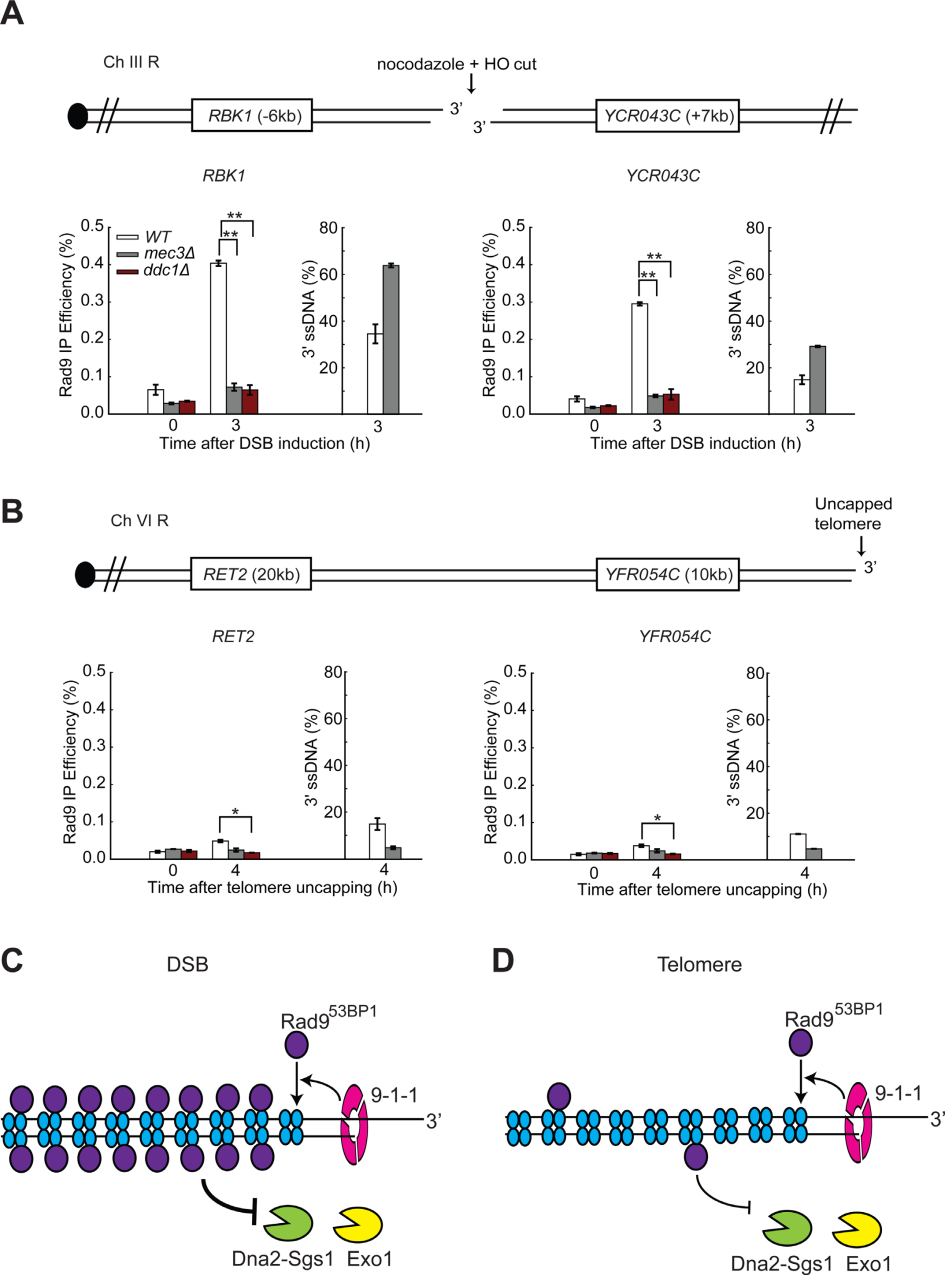
The 9-1-1 complex recruits more Rad9^53BP1^ near DSBs than uncapped telomeres. (**A, B**) ChIP analyses of Rad9-HA recruitment near DSBs (A) and uncapped telomeres (B). The ssDNA data were taken from Figure 1E and Supplementary Figure S1B. The data plotted and the *P*-values are as described in Figure 1. (**C, D**) The 9-1-1 complex stimulates the recruitment of Rad9^53BP1^ which inhibits Dna2-Sgs1 and Exo1, but Rad9^53BP1^ binds more poorly to uncapped telomeres.

To test the effect of 9-1-1 on the recruitment of Rad9^53BP1^, we performed ChIP experiments to examine the binding of Rad9^53BP1^ near sites of damage. Consistent with published results (34,44), we detected Rad9^53BP1^ recruitment to sites flanking the DSBs but not to a control locus, *PDA1*, 3 h following DSB induction (Figure 3A, Supplementary Figure S3B and SC). Importantly, *mec3Δ* and *ddc1Δ* strains each had much less Rad9^53BP1^ binding at these loci, showing that 9-1-1 does indeed contribute to the recruitment of Rad9^53BP1^ to DSBs. These ChIP data are consistent with the role of the clamp loader Rad24^RAD17^ in stimulating Rad9^53BP1^ recruitment to DSBs (44). As Rad9^53BP1^ is a strong inhibitor of DSB resection (33,34,45), the reduced Rad9^53BP1^ binding in *mec3Δ* and *ddc1Δ* strains (6–8 fold Figure 3A) could readily account for the hyper resection phenotype observed in *mec3Δ* and *ddc1Δ* strains at DSBs (Figure 1E).

One possibility why 9-1-1 had different effects on resection at telomeres versus DSBs was that Rad9^53BP1^ binds more poorly near telomeres compared to DSBs, perhaps due to the nature of the chromatin at telomeric and sub-telomeric regions. Rad9^53BP1^ binds chromatin via phosphorylated H2A serine 129 and methylated H3 lysine 79 and the levels of these chromatin modifications are affected near telomeres (46–48). To test the hypothesis that Rad9 binds at different levels, we performed ChIP experiments to examine Rad9^53BP1^ binding near uncapped telomeres and at DSBs (Figure 3B versus Figure 3A). To normalize for the amount of DNA damage, which might affect Rad9^53BP1^ binding, we examined loci and times with comparable levels of ssDNA (Figure 3A and B). In support of the hypothesis, we observed much less Rad9^53BP1^ recruitment to the telomeric *RET2* and *YFR054C* loci after telomere uncapping compared to the DSB loci, *RBK1* and *YCR043C* (compare wild type in Figure 3B and A). Even so, the binding of Rad9^53BP1^ near uncapped telomeres was reduced in *mec3Δ* and *ddc1Δ* mutants (Figure 3B, Supplementary Figure S3D), showing that 9-1-1 also contributes to the recruitment of Rad9^53BP1^ following telomere uncapping. Together our data suggest that 9-1-1 inhibits DSB resection by recruiting Rad9^53BP1^ (Figure 3C). However, at uncapped telomeres less Rad9^53BP1^ is recruited, permitting 9-1-1 to play a stimulatory role on resection (Figure 3D).

### The 9-1-1 complex stimulates DSB resection in *rad9Δ* cells

**Figure 4.**
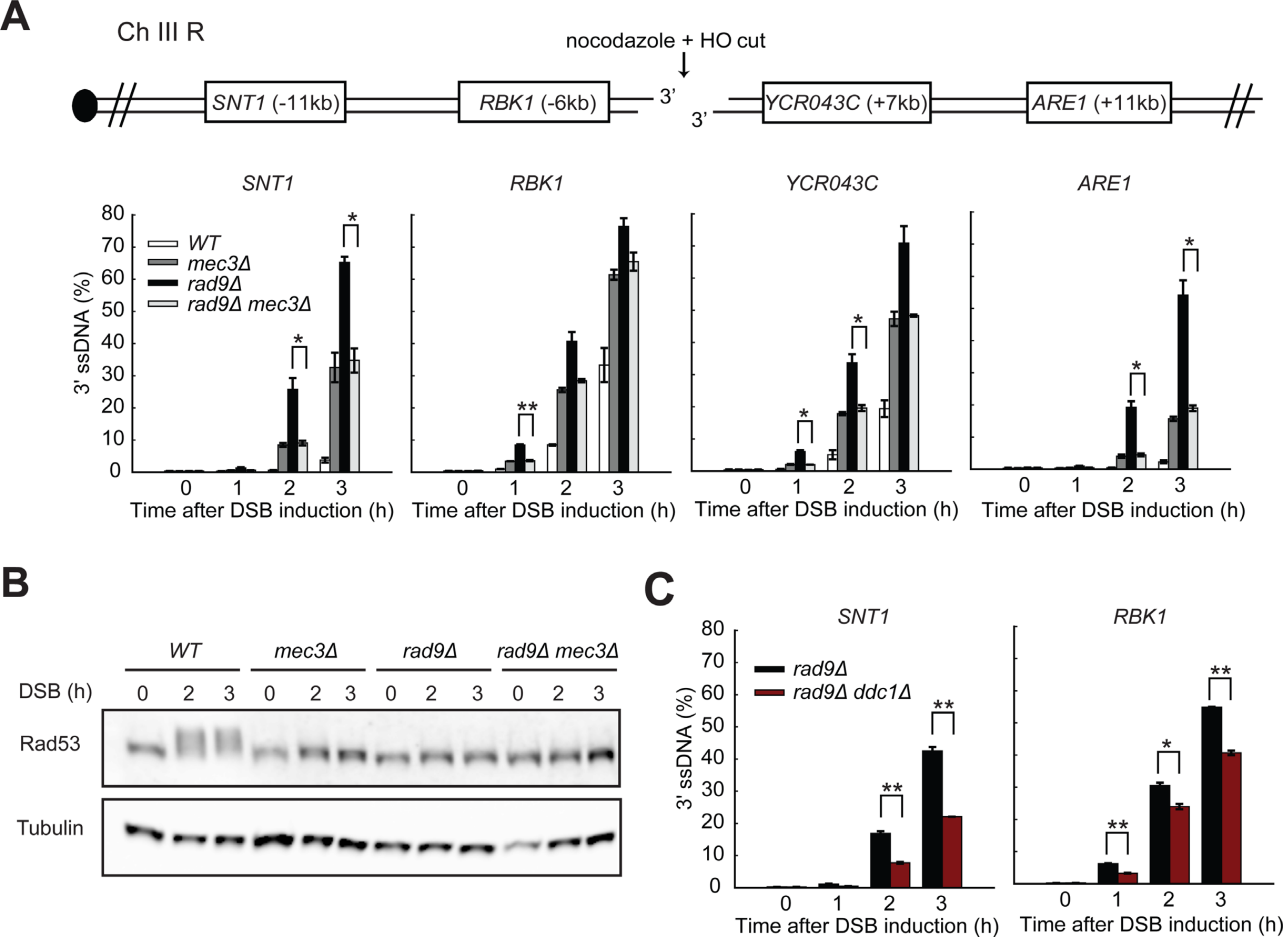
The 9-1-1 complex stimulates resection in *rad9Δ* cells. (**A,C**) Analysis of 3′ ssDNA accumulation in JKM179 strains at the indicated loci. The data plotted and the *P*-values are as described in Figure 1. (**B**) Yeast strains of the indicated genotypes were subjected to western blot analysis with anti-Rad53 and anti-tubulin antibodies following DSB induction.

To test the hypothesis that Rad9^53BP1^ affects 9-1-1-dependent resection, we examined resection at DSBs in the complete absence of Rad9^53BP1^. Consistent with results previously reported (33,34,45), Rad9^53BP1^ inhibited resection at DSBs, since *rad9Δ* strains showed greatly increased levels of 3′ ssDNA at all four loci examined (Figure 4A). Interestingly, *rad9Δ* caused a greater increase in ssDNA than *mec3Δ*, at all loci and all times examined (Figure 4A), showing that the effects of Rad9^53BP1^ were stronger. Importantly, we also observed that *rad9Δ mec3Δ* strains accumulated intermediate levels of ssDNA, more like *mec3Δ* than *rad9Δ* strains (Figure 4A). Thus, *mec3*Δ is epistatic to *rad9*Δ in this context. These data show that at DSBs, just as at uncapped telomeres (21,23,24), the very high levels of ssDNA that accumulate in *rad9Δ* strains are dependent on the 9-1-1 complex. The role of 9-1-1 seems to be particularly important in supporting extensive resection in *rad9Δ* strains as the effect of 9-1-1 is more obvious further from the break, at *SNT1* and *ARE1*, 11 kb from the break (Figure 4A).

We believe that the resection stimulatory role of 9-1-1 is independent of checkpoint signalling since *mec3Δ, rad9Δ* and *rad9Δ mec3Δ* mutants are all completely checkpoint defective as detected by the Rad53 phosphorylation assay (Figure 4B). We also conclude that the 9-1-1 complex *per se*, rather than Mec3 specifically, is important for stimulating resection in *rad9Δ* mutants because Ddc1, like its partner Mec3, stimulates resection (Figure 4C). Together, the results in Figures 3 and 4 suggest that 9-1-1 catalyses efficient Rad9^53BP1^ recruitment near DSBs and this inhibits resection. However, in *rad9Δ* strains, 9-1-1 stimulates resection at DSBs, just as at uncapped telomeres.

### The 9-1-1 complex stimulates Exo1- and Dna2-Sgs1-dependent resection in *rad9Δ* cells

**Figure 5.**
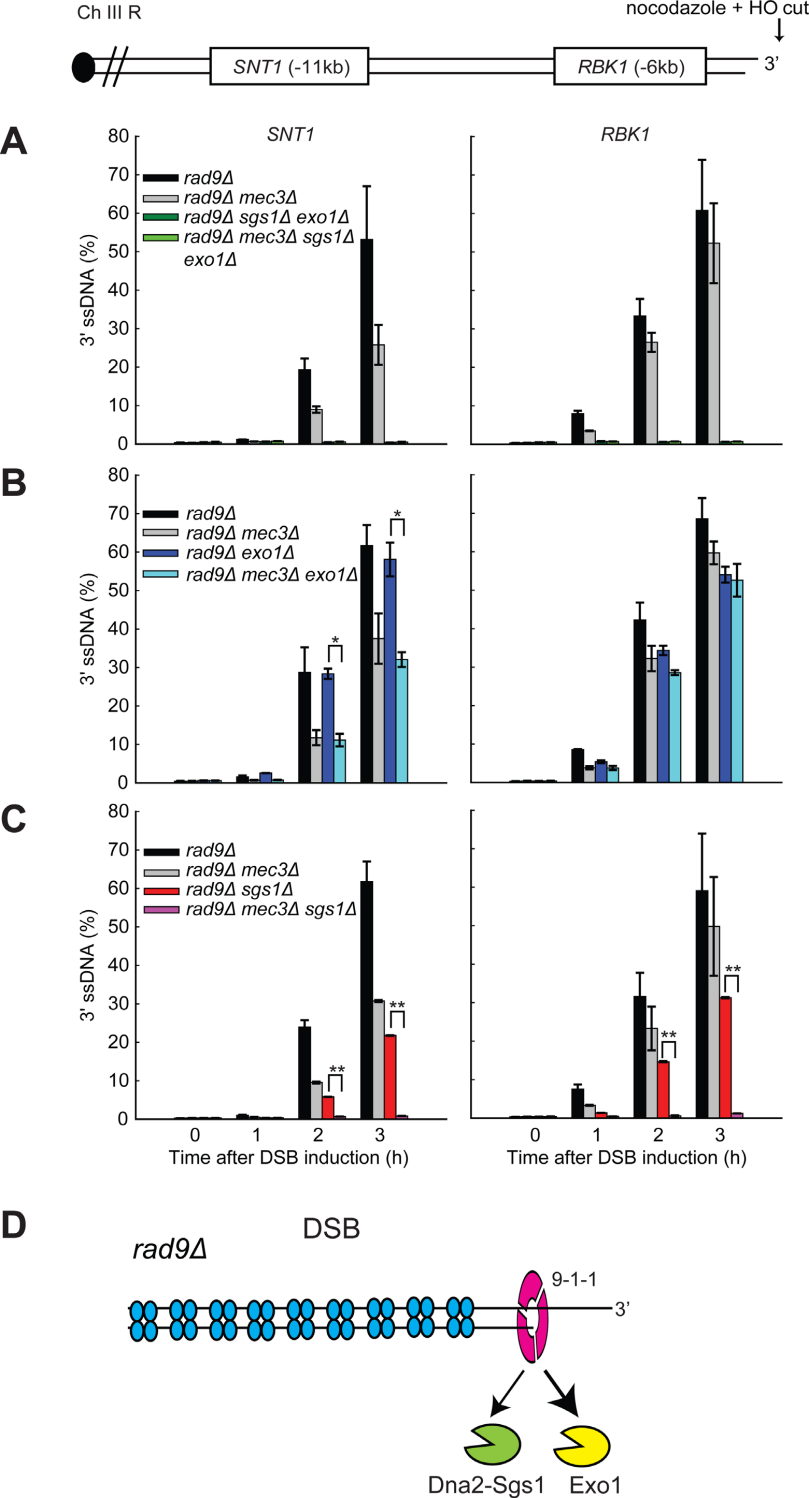
The 9-1-1 complex stimulates both Exo1- and Dna2-Sgs1-dependent resection in *rad9Δ* cells. (**A-C**) Analysis of 3′ ssDNA accumulation in *rad9Δ* background strains at the indicated loci. The data plotted and the *P*-values are as described in Figure 1. (**D**) The 9-1-1 complex stimulates both Exo1 and Dna2-Sgs1 in *rad9Δ* cells.

The 9-1-1 complex promotes resection at uncapped telomeres *in vivo*, where Rad9^53BP1^ binding is low (Figure 3B), and *in vitro* (in the absence of Rad9^53BP1^) by stimulating both Dna2-Sgs1- and Exo1-dependent resection (24). To test whether 9-1-1 stimulates Dna2-Sgs1- and Exo1-dependent resection at DSBs *in vivo*, we deleted *MEC3* in *rad9Δ exo1Δ, rad9Δ sgs1Δ, rad9Δ dna2Δ* or *rad9Δ sgs1Δ exo1Δ* genetic backgrounds and measured ssDNA accumulation (Figure 5A–C, Supplementary Figure S4). In agreement with the results in Figure 4A, *mec3Δ* decreased resection in all *rad9Δ* strains following the induction of DSBs (Figure 5A–C, compare *rad9Δ* and *rad9Δ mec3Δ* at *SNT1*). Consistent with the results in *RAD9^+^* strains, deletion of *EXO1* or *SGS1*/*DNA2* reduced resection in *rad9Δ* strains and deletion of both *SGS1* and *EXO1* completed eliminated resection (Figure 5A–C, Supplementary Figure S4). We conclude that all resection in *rad9Δ* strains is dependent on Dna2-Sgs1 or Exo1. This suggests that other nucleases, such as MRX-Sae2, do not become hyperactive in the absence of Rad9^53BP1^.

Interestingly, resection in *rad9Δ* strains (as in *mec3Δ* strains, Figure 2) appears to be more dependent on Dna2-Sgs1 than Exo1 (compare *rad9Δ exo1Δ* strains in Figure 5B with *rad9Δ dna2Δ* strains in Figure 5C, and in Supplementary Figures S5 and S6). This contrasts to what is observed in *WT* strains where resection is more or less equally dependent on Exo1 or Dna2-Sgs1 (Figure 2). These comparisons suggest that Rad9^53BP1^, like 9-1-1 (Figure 2), inhibits Dna2-Sgs1 more than Exo1.

To determine whether 9-1-1 promotes Dna2-Sgs1-dependent resection in the absence of Rad9^53BP1^, we examined how *mec3Δ* affects ssDNA accumulation in *rad9Δ exo1Δ* strains, where all resection is due to Dna2-Sgs1. It was clear that the *mec3Δ* mutation reduced resection in *rad9Δ exo1Δ* strains (Figure 5B, compare *rad9Δ mec3Δ exo1Δ* with *rad9Δ exo1Δ* strains at *SNT1*). The effect of 9-1-1 on Dna2-Sgs1 is more clearly seen at loci further away from the breaks likely because in the absence of 9-1-1, Sgs1-Dna2 can still resect DNA, but not as efficiently. We conclude that 9-1-1 facilitates Dna2-Sgs1-dependent resection at DSBs in *rad9Δ* strains.

To determine whether 9-1-1 promotes Exo1-dependent resection in the absence of Rad9^53BP1^, we examined how *mec3Δ* affects ssDNA accumulation in *rad9Δ sgs1Δ* or *rad9Δ dna2Δ* strains, where all resection is due to Exo1. Similar to the results obtained in *RAD9+ sgs1Δ* or *RAD9+ dna2Δ* strains (Figure 2C, Supplementary Figure S3A), the *mec3Δ* mutation completely eliminated resection in *rad9Δ sgs1Δ* or *rad9Δ dna2Δ* strains (Figure 5C, compare *rad9Δ mec3Δ sgs1Δ* with *rad9Δ sgs1*Δ strains, Supplementary Figure S4, compare *rad9Δ mec3Δ dna2Δ* with *rad9Δ dna2*Δ strains), confirming that 9-1-1 is critical for Exo1-dependent resection in *rad9Δ* strains. The observation that *rad9Δ mec3Δ sgs1*Δ or *rad9Δ mec3Δ dna*2Δ strains had much less ssDNA than *rad9Δ mec3Δ exo1*Δ strains (Figure 5B and C, Supplementary Figure S5 and S6) shows that Exo1 is more dependent on 9-1-1 activity than Dna2-Sgs1. We conclude that the 9-1-1 complex stimulates both Dna2-Sgs1- and Exo1-dependent resection, but that Exo1 is more dependent on 9-1-1 (Figure 5D).

### The 9-1-1 complex affects the binding of both Exo1 and Dna2-Sgs1 to DNA

**Figure 6.**
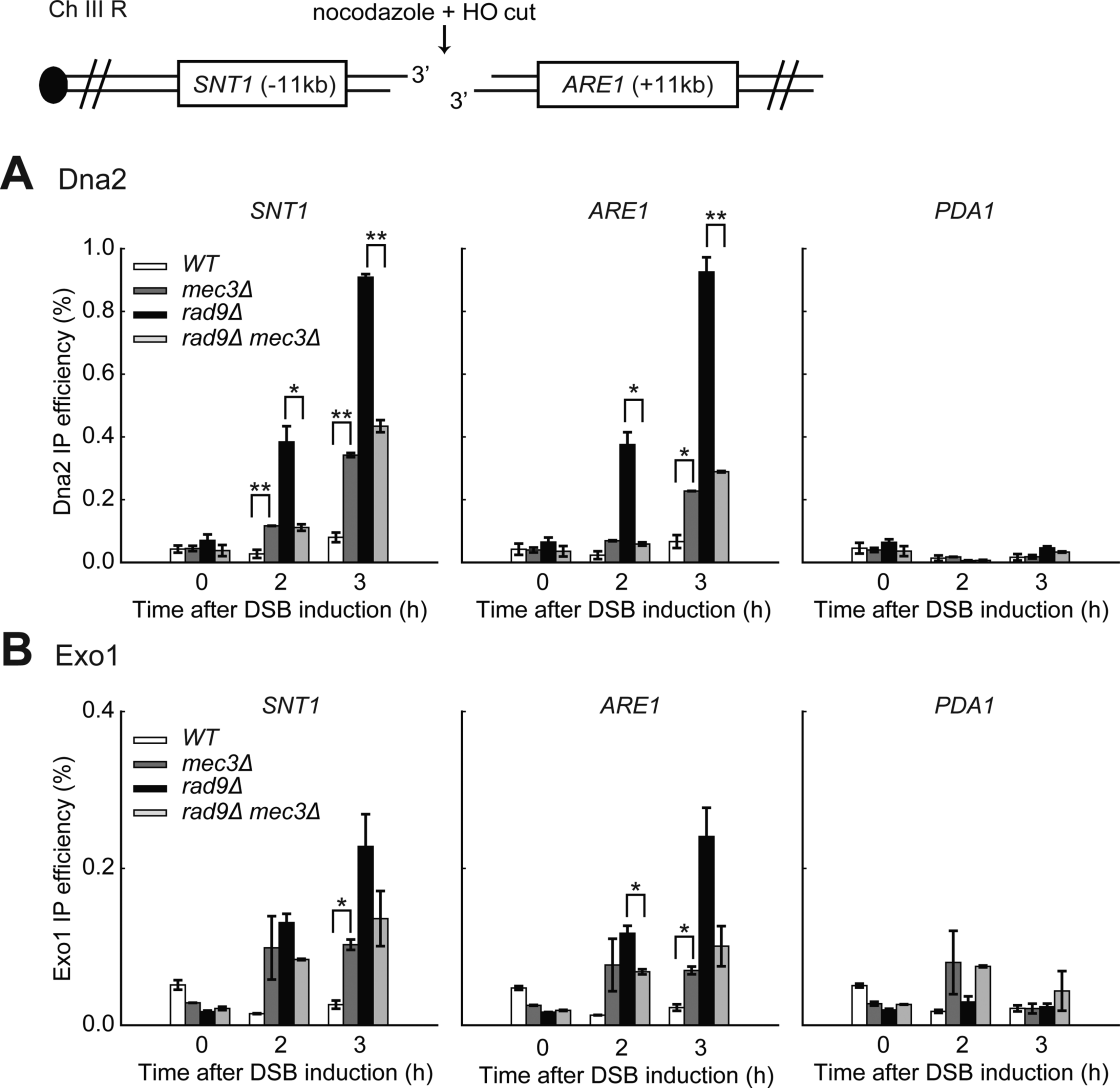
The 9-1-1 complex affects the binding of both Exo1 and Dna2-Sgs1 to DNA. (**A,B**) ChIP analysis of Dna2-Myc and Exo1-Myc recruitment to DSBs in JKM179 background strains. The data plotted and the *P*-values are as described in Figure 1.

To better understand how the 9-1-1 complex and Rad9^53BP1^ affect resection by Dna2-Sgs1 and Exo1, we examined the effects of Mec3 and Rad9^53BP1^ on the binding of Dna2 and Exo1 near DSB sites (Figure 6A and B). We found that *rad9Δ* strongly increased the binding of Dna2 and Exo1 to *ARE1* and *SNT1* near a DSB, but not to a control locus *PDA1* (compare WT and *rad9Δ*, Figure 6A and B). This observation suggests that Rad9^53BP1^ inhibits the binding of both Dna2 and Exo1 to DSBs. *mec3Δ* also increased binding of Dna2 and Exo1 to *ARE1* and *SNT1* (compare *mec3Δ* and WT, Figure 6A and B), likely due to decreased Rad9^53BP1^ recruitment to DSBs (Figure 3A). Importantly, *rad9Δ mec3Δ* strains had reduced binding of Dna2 and Exo1 to these DSB loci compared to *rad9Δ* strains (Figure 6A and B, Supplementary Figure S7A), suggesting that 9-1-1 stimulates Dna2-Sgs1 and Exo1 recruitment (and resection) in *rad9Δ* cells.

Our previous biochemical studies showed that the 9-1-1 complex directly stimulates the activities of DNA2 and EXO1 (24). Consistent with this hypothesis, we observed increased binding of Mec3 and Ddc1 (the two subunits of the 9-1-1 complex) near DSBs in *rad9Δ* strains (Supplementary Figure S7B and SC), which explains the increased recruitment of Dna2 and Exo1 in this mutant background. Collectively, the data in Figure 6 suggest that the 9-1-1 complex affects Dna2-Sgs1 and Exo1-dependent resection by regulating the recruitment of these nuclease and helicase activities near DSBs.

### The 9-1-1 complex coordinates DSB resection at another internal locus

We find that the 9-1-1 complex stimulates resection at an HO-induced DSB but other groups have reported that 9-1-1 showed no effect or inhibited resection (25,26). Therefore to clarify the role of 9-1-1 in DSB resection, we examined resection after DSB induction at another locus in a different chromosome and in a different genetic background. We used a strain where an HO DSB can be induced at the *URA3* locus on chromosome V (26). As before, we performed all the experiments in the presence of nocodazole to ensure that any effects we observed were not affected by cell cycle position. We found that at *URA3*, in W303 strains, DSBs were induced with lower efficiencies compared to the *MAT* locus in JKM179 strains, reaching just over 60% in all strains 1 h after addition of galactose, and 90% after 3 h (compare Supplementary Figure S7D with Figure 1). However, *mec3Δ* did not affect DSB formation (Supplementary Figure S7D).

**Figure 7.**
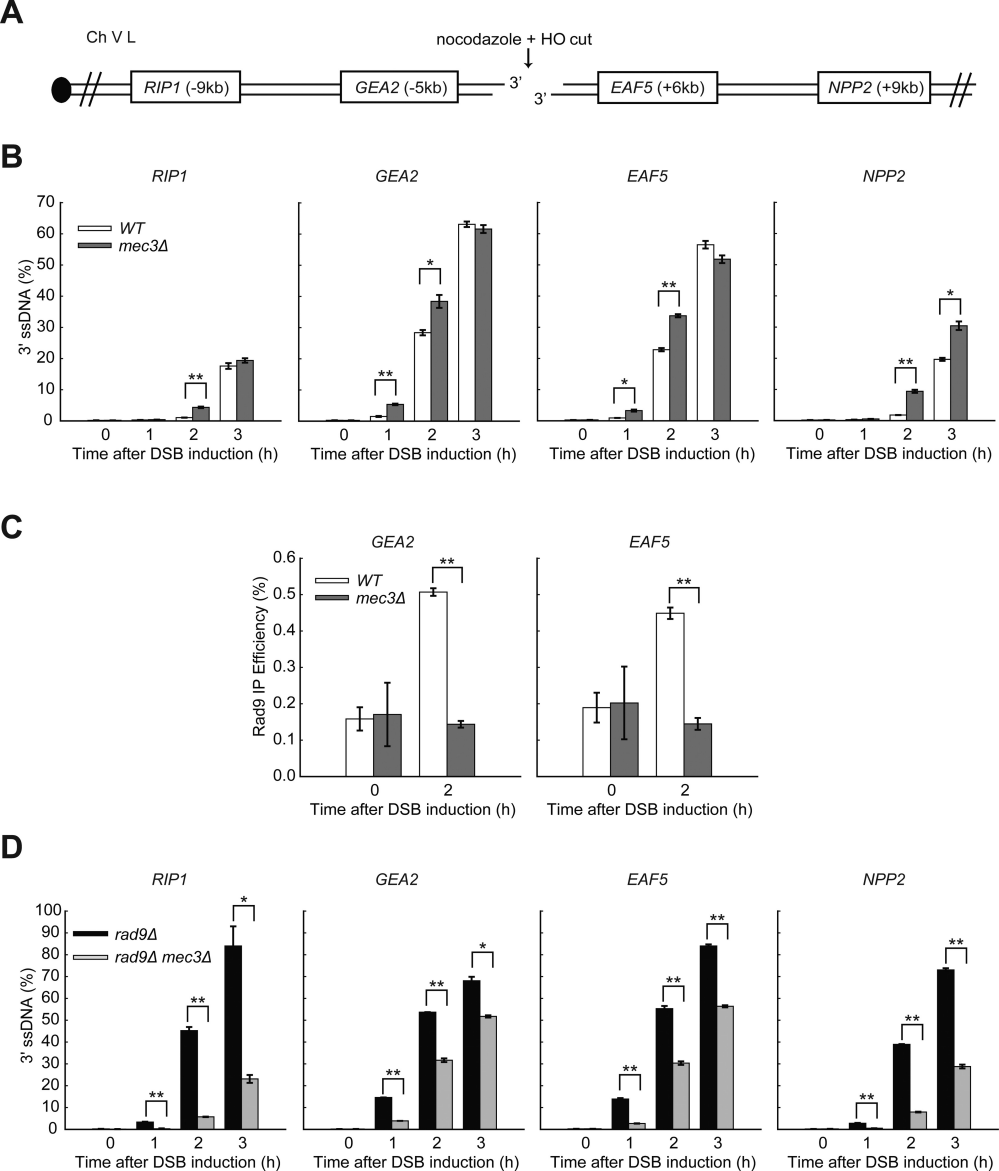
The 9-1-1 complex coordinates DSB resection at the *URA3* locus. (**A**) Map of chromosome V showing an HO endonuclease site and loci examined in this study. (**B,D**) Analysis of 3′ ssDNA accumulation in the indicated strains. (**C**) ChIP analyses of Rad9-HA recruitment near DSBs. All the experiments were performed in nocodazole arrested cells. The data plotted and the *P*-values are as described in Figure 1.

To examine resection, we measured ssDNA accumulation at loci on both sides of the break using QAOS (Figure 7A). Interestingly, we found that resection at *URA3* generated more ssDNA (60% at 5 kb after 3 h in the wild-type strains) than at the *MAT* locus (35% at 6 kb after 3 h), possibly suggesting that a higher fraction of breaks were resected or resected at fast rates at the *URA3* locus in W303 strains. Importantly, in our hands, *mec3Δ* strains consistently showed higher levels of ssDNA compared to the wild type (Figure 7B). This result is consistent with our finding at the *MAT* locus (Figure 1), but contradicts Aylon and Kupiec (26), who showed that *rad24Δ* mutants, which cannot load the 9-1-1 complex on DNA, had less resection. We believe this difference arises likely because Aylon and Kupiec performed their resection experiments in asynchronous cultures, and *rad24Δ* mutants, which are checkpoint deficient, escape G2/M arrest and enter G1, where resection is less active due to lower CDK1 activity. We conclude that the 9-1-1 complex inhibits DSB resection at the *URA3* locus.

To determine whether 9-1-1 inhibits DSB resection at the *URA3* locus via recruitment of Rad9^53BP1^, we examined Rad9^53BP1^ binding to *GEA2* and *EAF5* by ChIP. Consistent with our results at the *MAT* locus, DSB induction induced enrichment of Rad9^53BP1^ near DSBs, and recruitment is dependent on Mec3 (Figure 7C, Supplementary Figure S7E). We conclude that 9-1-1 inhibits resection at URA3 by affecting Rad9^53BP1^ recruitment near DSBs.

Finally, we tested whether 9-1-1 has the ability to stimulate DSB resection in *rad9Δ* mutants (Figure 7D). Importantly, *rad9Δ mec3Δ* accumulated much less ssDNA than *rad9Δ* strains (Figure 7D), showing that 9-1-1 strongly stimulates DSB resection at *URA3*. We conclude that 9-1-1 both inhibits and stimulates DSB resection at the *URA3* locus, as well as at the *MAT* locus, and presumably does at most non-telomeric DSB loci.

## DISCUSSION

ssDNA is a critical intermediate in the DNA damage response because it is required for homologous recombination and DNA damage checkpoint maintenance. In all eukaryotic cells two major nucleases, Exo1 and Dna2-Sgs1, contribute to long-range resection *in vivo*. Why two nucleases, rather than one, are required and how they are coordinated remains unclear. One hypothesis to explain the use of two nucleases is that each nuclease has preference for different types of chromatin substrate and the nucleases interchange to ensure the most efficient control over resection. Since ssDNA is potentially very harmful to cells, there is clear need to regulate Exo1 and Dna2-Sgs1 to ensure that sufficient ssDNA is generated to help the DNA damage response, but not too much to harm cell survival.

A number of DDR proteins regulate DNA resection. Tel1 and its mammalian homologue ATM stimulate resection initiation by activating MRX and CtIP (8,49,50). Mec1^ATR^ also stimulates resection but this mechanism is poorly understood (22,34). Other DDR proteins inhibit resection, presumably reducing the harmful effects of ssDNA on genome stability. Rad9^53BP1^ and its mammalian homologue 53BP1 inhibit resection (21,51). Mec1^ATR^ inhibits resection in yeast by at least two mechanisms: (i) stimulating Rad53-dependent phosphorylation of Exo1 which leads to downregulation of Exo1 activity (15,16) and (ii) promoting the binding of the resection inhibitor Rad9^53BP1^ close to DNA lesions (34).

**Figure 8.**
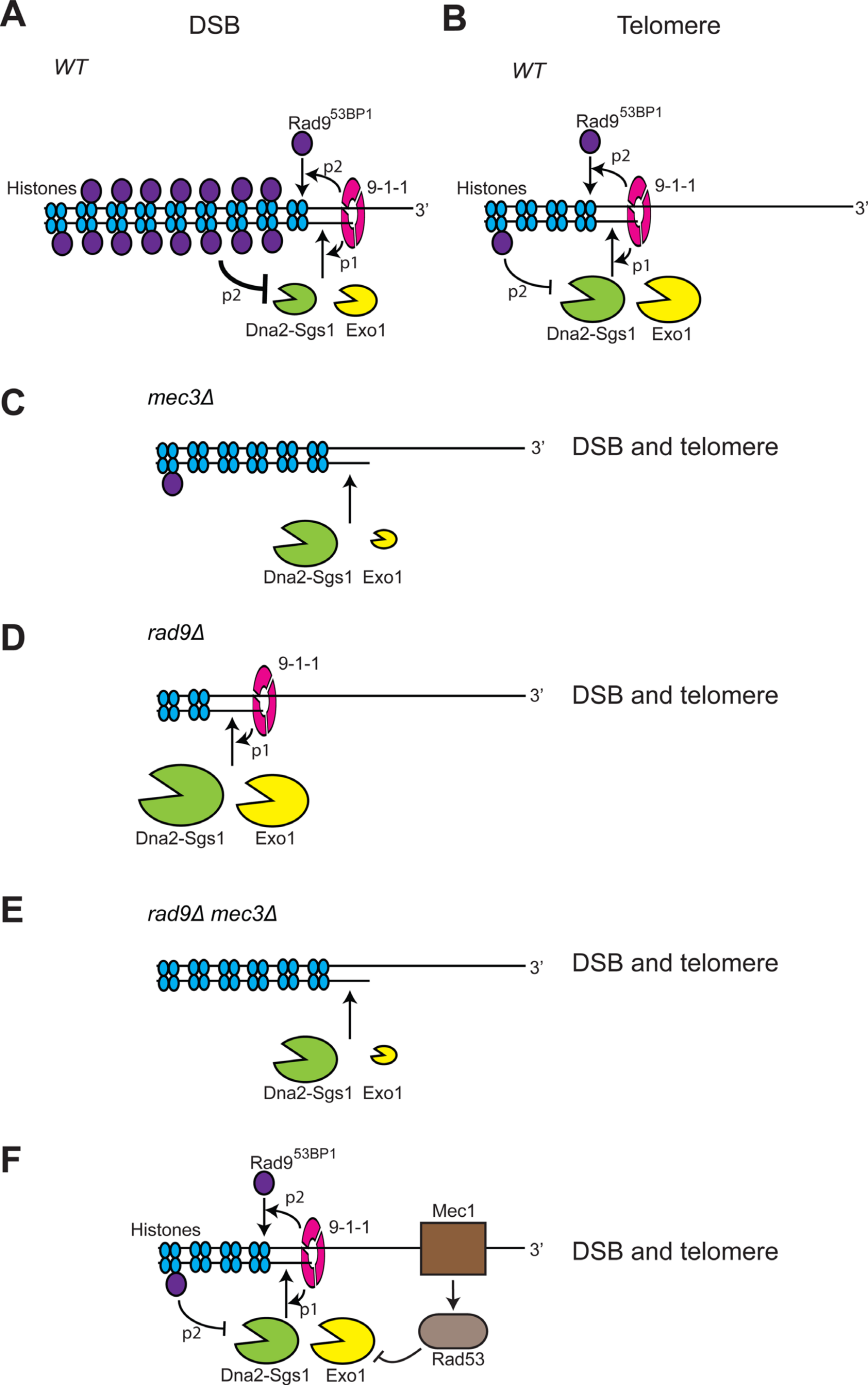
Control of resection at DSBs and telomeres. Models for the roles of 9-1-1, Rad9^53BP1^, Exo1 and Dna2-Sgs1 on resection near DSBs and at uncapped telomeres. The size of the nucleases in each schematic indicates relative resection activities and is deduced by ssDNA measurements in the different genetic settings. Data supporting these figures are taken from Figures 1–7 and Ngo *et al*. (24). (**A, B**) 9-1-1 stimulates recruitment of Exo1 and Dna2-Sgs1 to facilitate resection (pathway 1, p1). 9-1-1 stimulates recruitment of Rad9^53BP1^ to inhibit resection (pathway 2, p2). Rad9^53BP1^ binds more near DSBs than uncapped telomeres. (**C**) In *mec3Δ* cells, there is less Rad9^53BP1^ recruitment (lack of p2), but there is no 9-1-1 to stimulate activity of Exo1 and Dna2-Sgs1 (lack of p1). At DSBs Exo1 is less active (lack of p1) but Dna2-Sgs1 is more active (lack of p2) (C compared to A). The overall effect is increased resection in *mec3*Δ mutants (C compared to A). At telomeres Exo1 is less active (lack of p1) but Dna2-Sgs1 activity remains little changed because little Rad9^53BP1^ binds (p2 less active at telomeres), and so the overall effect of *mec3*Δ is reduced resection (C compared to B). (**D**) In *rad9Δ* cells, there is no Rad9^53BP1^ recruitment. Therefore, Dna2-Sgs1 and Exo1 are more active than in (A, B). Dna2-Sgs1 is more active than Exo1 in the absence of Rad9^53BP1^. (**E**) In *rad9Δ mec3Δ* cells, there is no 9-1-1 to stimulate Exo1 or Dna2-Sgs1. Therefore, Dna2-Sgs1 and Exo1 are less active than in D. Exo1 is more dependent on 9-1-1 than Dna2-Sgs1. (**F**) Mec1^ATR^ also initiates a checkpoint cascade to inhibit Exo1.

Here we establish the central role of the 9-1-1 complex in coordinating Exo1 and Dna2-Sgs1 activities. Figure 8A and B illustrate the roles of the 9-1-1 complex, Rad9^53BP1^, Exo1 and Dna2-Sgs1 at DSBs and uncapped telomeres. We propose that at DSBs and uncapped telomeres, 9-1-1 helps recruit Exo1 and Dna2-Sgs1 to DNA to facilitate resection (Pathway 1, P1). 9-1-1 also helps recruit Rad9^53BP1^, a resection inhibitor to inhibit resection (Pathway 2, P2) (33,45). We suggest that the essential difference between DSBs and uncapped telomeres is due to the balance between Pathways 1 and 2 at telomeres versus internal chromosomal loci, with Pathway 2, the recruitment of Rad9^53BP1^, working much better at internal DSBs versus telomeres. This difference likely explains why resection at uncapped telomeres (8 kb/h) is faster than at DSBs (3.5 kb/h), as well as explains the different effects of 9-1-1 on resection at telomeres versus DSBs.

Interestingly, in the absence of 9-1-1 (*mec3Δ* cells), Rad9^53BP1^, or both, resection at DSBs and uncapped telomeres is similar (Figure 8C–E). In *mec3Δ* cells, at both types of loci, there is less Rad9^53BP1^ recruitment (lack of Pathway 2) and there is no 9-1-1 to stimulate activity of Exo1 and Dna2-Sgs1 (lack of Pathway 1; Figure 8C). The overall effect of 9-1-1 inactivation is increased resection at DSBs but decreased resection at uncapped telomeres (compare Figure 8C with Figure 8A and B). At DSBs, in *mec3Δ* strains, Exo1 is less active due to lack of Pathway 1 but Dna2-Sgs1 more than compensates for this, and is more active, due to lack of Pathway 2 (compare Figure 8C with Figure 8A). At telomeres, in *mec3Δ* strains, Exo1 is less active (lack of Pathway 1), but in contrast to at DSBs, Dna2-Sgs1 activity remains little changed, because Pathway 2 is less active near uncapped telomeres (compare Figure 8C with Figure 8B). Thus, the overall effect of loss of 9-1-1 at uncapped telomeres is reduced resection. Importantly, we find that the 9-1-1 complex strongly stimulates resection at both DSBs and uncapped telomeres and this is most clearly seen in the absence of Rad9^53BP1^ (compare Figure 8D and Figure 8E).

We are unclear why Rad9^53BP1^ binds less well near uncapped telomeres but this could be due to the nature of the chromatin at telomeric and sub-telomeric regions with reduced methylation of H3 lysine 79, which is needed for Rad9^53BP1^ binding (47). Alternatively, there might be insufficient Rad9^53BP1^ available to bind all the uncapped telomeres in comparison with the smaller number of ends induced by a single DSB. Irrespective of the reason, the weak binding of Rad9^53BP1^ to telomeres appears to be conserved, since Crb2, the Rad9^53BP1^ orthologue in fission yeast, also binds less efficiently near uncapped telomeres than DSBs (52). Even though Rad9^53BP1^ binds weakly to telomeres, it still inhibits resection since *rad9Δ* strains clearly show increased resection at uncapped telomeres (and DSBs; Figure 8D) (21,23,24).

Interestingly, all our experiments suggest that Exo1 is more reliant on 9-1-1 than Dna2-Sgs1 for its activity *in vivo*. Furthermore, Rad9^53BP1^ inhibits Dna2-Sgs1 at DSBs more than Exo1, consistent with a recent study (53). Thus, it seems that the combined effects of the checkpoint sliding clamp and the Rad9^53BP1^ checkpoint mediator protein are to stimulate Exo1 and inhibit Dna2-Sgs1-dependent nuclease activities. Why DNA damage checkpoint proteins should favour Exo1 over Dna2-Sgs1 is unclear. However, we note that Exo1 is also targeted and inhibited by DNA damage checkpoint kinase cascades (Figure 8F) (15,16).

We propose that the ring-shaped 9-1-1 clamp recruits and tethers Exo1 to DNA, and that tethered Exo1 is better able to resect through Rad9^53BP1^ containing chromatin. We suggest that 9-1-1 also recruits Dna2-Sgs1 to damaged sites but that Dna2-Sgs1 activity (presumably tethered on DNA by the hexameric Sgs1 helicase ring) is more active on Rad9^53BP1^ free chromatin. Thus, 9-1-1 may play a critical role in promoting the interchange of nucleases to facilitate resection through different types of chromatin. It has also been reported that Dna2-Sgs1 and Exo1 are recruited to DSBs by the MRX^MRN^ complex and replication protein A (RPA) (54,55). However, MRX dissociates from the ends following extensive resection (56). Thus, the recruitment role of 9-1-1 would appear to be more important at sites distal to the initial lesion.

Overall, our experiments reinforce the idea that 9-1-1 checkpoint sliding clamp binding to DNA lesions modulates DNA damage metabolism as well as stimulating checkpoint-dependent cell cycle arrest. We suggest that whether 9-1-1 promotes or inhibits resection is regulated by type of chromatin found near the DNA damage and other factors such as the activity of the central checkpoint kinase Mec1^ATR^. Mec1^ATR^-dependent phosphorylation of Ddc1 subunit of 9-1-1 stimulates the interaction of 9-1-1 with Dpb11^TopBP1^, which in turn interacts with Rad9^53BP1^ and Mec1^ATR^ to help activate the DNA damage checkpoint (57,58). We suggest that Mec1^ATR^ phosphorylation of Ddc1 may switch the role of 9-1-1 from stimulating resection (Pathway 1) to facilitating Rad9^53BP1^ recruitment and checkpoint activation (Pathway 2) (57), and therefore reduce the potential harm caused by excess ssDNA accumulation.

Although we have found different roles for 9-1-1, Rad9^53BP1^, Exo1 and Dna2-Sgs1 at DSBs in comparison to uncapped telomeres, we do not consider the two loci to be distinct classes. In reality, we imagine that there is a spectrum of locus types between, and perhaps beyond, the DSB-like and telomere-like loci we have examined. The loci will differ, for example, in their affinity for Rad9^53BP1^. It is interesting that Rad9^53BP1^ has recently been reported to constitutively bind chromatin in combination with Aft1 transcription factor, and to bind with preference to fragile genomic regions (59). This pattern of binding is consistent with a role for Rad9^53BP1^, limiting resection at those genomic locations that are more likely to suffer damage. In contrast, at telomeres, it is perhaps useful if cells favour resection because homologous recombination between the repetitive, non-coding telomeric DNAs is essentially harmless, whereas the alternative NHEJ pathway of repair is potentially much more dangerous because it could induce dicentric chromosomes and genetic instability.

In conclusion, our findings reveal the central role of the 9-1-1 checkpoint sliding clamp coordinating DNA resection in addition to its better known role of stimulating cell cycle arrest. 9-1-1 recruits both an inhibitor (Rad9^53BP1^) and two activators of resection (Exo1 and Sgs1/Dna2) to DSBs. It is clear that mammalian 53BP1, like its yeast orthologue Rad9^53BP1^, inhibits resection (51). It will be interesting to see if the 9-1-1 complex regulates DSB resection *in vivo* in mammalian cells and whether this role contributes to the involvement of 9-1-1 in processes like recombination and cancer progression (60–62). It seems reasonable to assume that control over resection in mammalian cells will be at least as sophisticated as in yeast and that the 9-1-1 complex is a central player in the DNA damage response network across all eukaryotic cells.

## SUPPLEMENTARY DATA

Supplementary Data are available at NAR Online.

SUPPLEMENTARY DATA
