# Supplementary material for: The 9-1-1 checkpoint clamp coordinates resection at DNA double strand breaks
Source: Nucleic Acids Res. 2015 Apr 29;43(10):5017–32. doi: 10.1093/nar/gkv409 (PMC4446447; doi:10.1093/nar/gkv409)
Supplement: SUPPLEMENTARY DATA [file supp_43_10_5017__index.html]

The 9-1-1 checkpoint clamp coordinates resection at DNA double strand breaks — The 9-1-1 checkpoint clamp coordinates resection at DNA double strand breaks — SUPPLEMENTARY DATA 

# The 9-1-1 checkpoint clamp coordinates resection at DNA double strand breaks

## SUPPLEMENTARY DATA

**Files in this Data Supplement:**

- SUPPLEMENTARY DATA
