# Supplementary material for: The 9-1-1 checkpoint clamp coordinates resection at DNA double strand breaks
Source: Nucleic Acids Res. 2015 Apr 29;43(10):5017–32. doi: 10.1093/nar/gkv409 (PMC4446447; doi:10.1093/nar/gkv409)
Supplement: SUPPLEMENTARY DATA [file supp_gkv409_nar-03105-d-2014-File010.pdf]

SUPPLEMENTARY FIGURE LEGENDS

Figure S1.

(A,B) Analysis of 3'ssDNA accumulation following DSB induction (A) and telomere uncapping (B) at the indicated loci. The data plotted are the means and the range from two strains (data from B taken from Ngo et al, 2014).

Figure S2.

Analysis of 3'ssDNA accumulation following telomere uncapping (at 32°C and 34°C) in the presence of nocodazole. The data plotted are the means from one strain.

Figure S3.

(A) Analysis of 3'ssDNA accumulation in JKM179 strains at the indicated loci. (N.B. all strains were in a *pif1Δ* background to suppress the lethality caused by deleting *DNA2*). (**B**) ChIP analyses of Rad9-HA recruitment at the indicated loci near DSBs. (**C-D**) ChIP analyses of Rad9-HA recruitment to control loci after DSB induction (C) and telomere uncapping (D). The data plotted are the means and the range from two strains. P values were calculated using unpaired two-tailed T test. * represents $P< 0.05$, ** represents $P< 0.01$.

Figure S4.

Analysis of 3'ssDNA accumulation in *rad9*∆ background strains at the indicated loci. All strains have *pif1*∆ mutations. The data plotted and the p values are as described in Figure S3.

Figure S5.

3' ssDNA accumulation in the indicated strains relative to wild type (data from Figure 2,4,5). The data plotted are the means and the range from two strains.

Figure S6.

3' ssDNA accumulation in the indicated strains relative to wild type (data from Figure 2,4,5). The data plotted are the means and the range from two strains.

Figure S7.

(A) ChIP analyses of Exo1-Myc recruitment to the indicated loci relative to a control locus following DSB induction in JKM179 background strains. The data plotted and the p values are as described in Figure S3. **(B,C)** ChIP analysis of Mec3-Myc and Ddc1-Myc recruitment to DSBs in JKM179 background strains. The data plotted are the means from one strain. **(D)** The efficiencies of DSB induction in the indicated strains at the *URA3* locus. (**E**) ChIP analyses of Rad9-HA recruitment at a control locus after DSB induction. The data plotted in D,E are the means and the range from two strains.

Table S1. Yeast strains used in the study.

Table S2. Yeast strains used in the study.

Table S3. DNA oligonucleotides used in the study.

Table S4. DNA oligonucleotides used in the study.

Figure S1

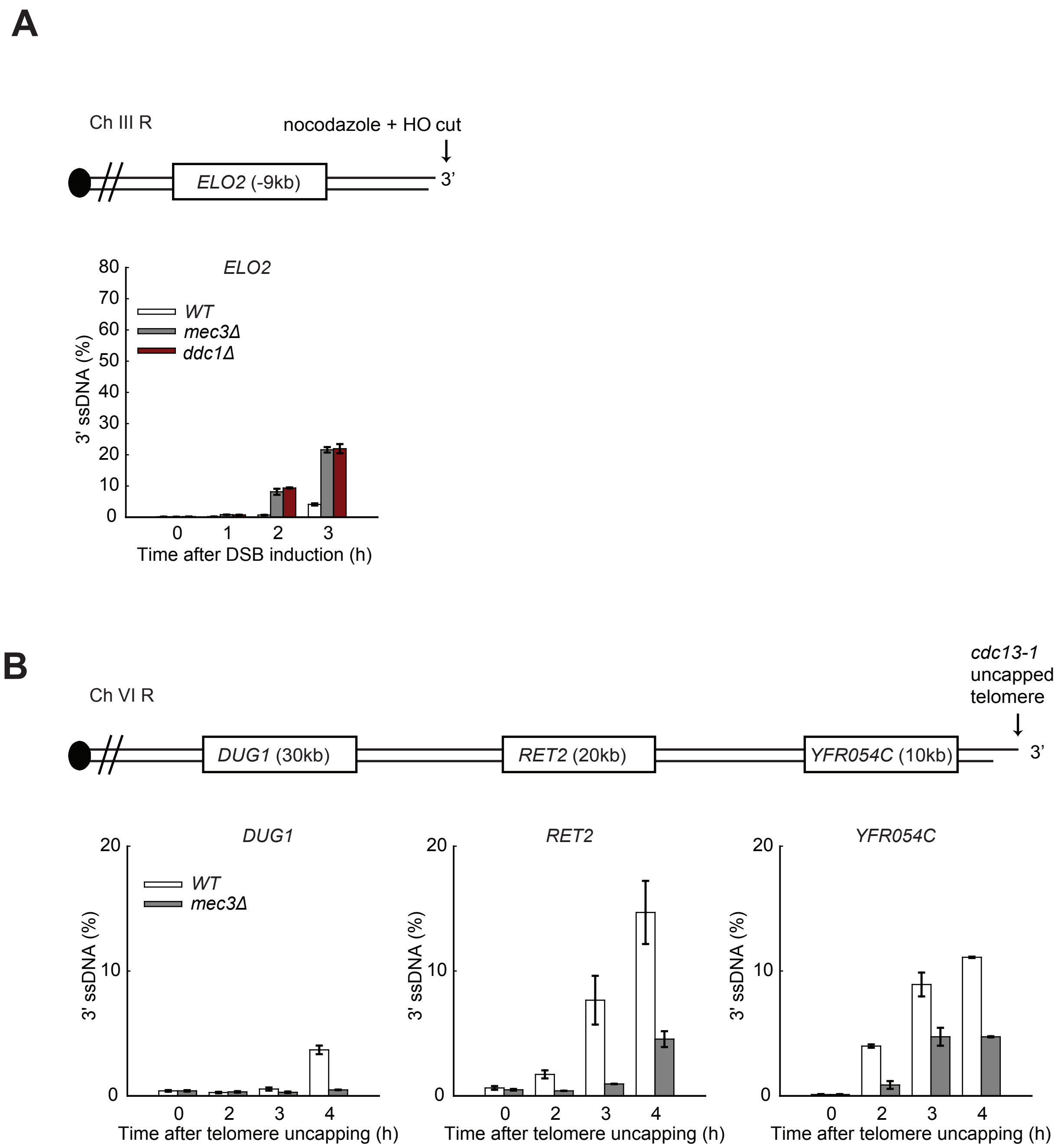
A
Ch III R
nocodazole + HO cut
3’
ELO2 (-9kb)
ELO2
WT
mec3Δ
ddc1Δ
3’ ssDNA (%)
0
10
20
30
40
50
60
70
80
0
1
2
3
Time after DSB induction (h)
B
Ch VI R
cdc13-1
uncapped
telomere
3’
DUG1 (30kb)
RET2 (20kb)
YFR054C (10kb)
DUG1
RET2
YFR054C
WT
mec3Δ
3’ ssDNA (%)
0
10
20
0
2
3
4
Time after telomere uncapping (h)

Figure S2

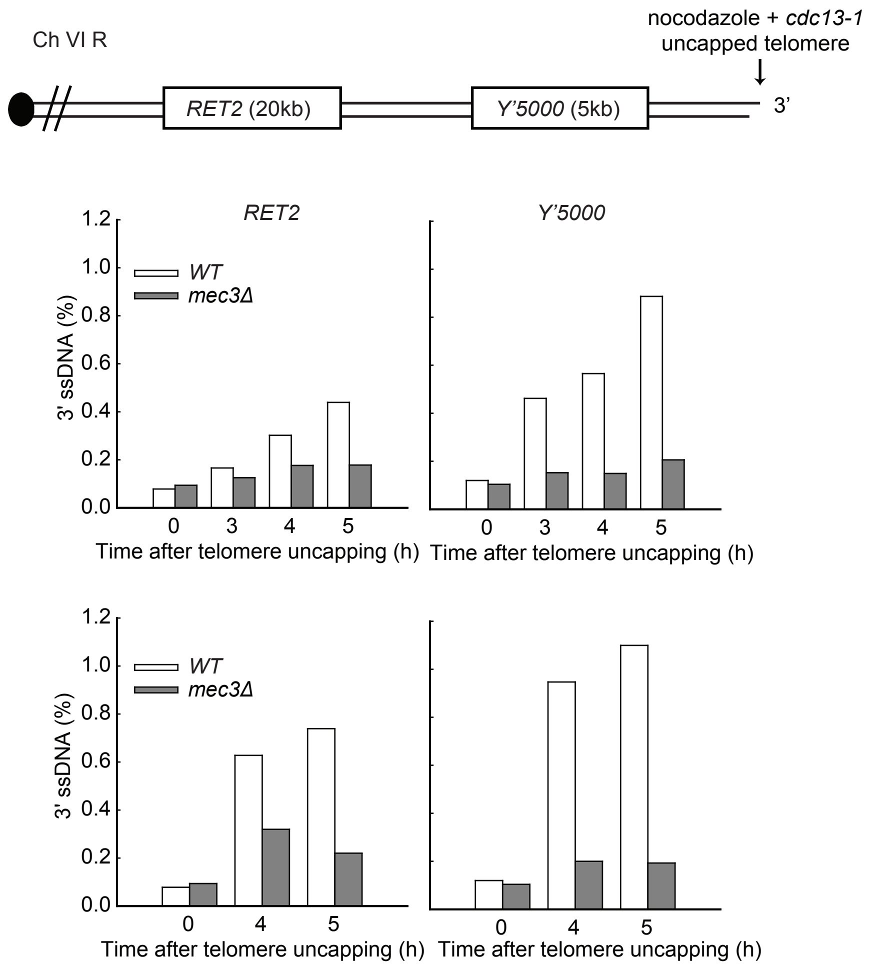
nocodazole + cdc13-1
uncapped telomere
Ch VI R
RET2 (20kb)
Y'5000 (5kb)
3'
32°C
RET2
Y'5000
1.2
1.0
0.8
0.6
0.4
0.2
0.0
WT
mec3Δ
3' ssDNA (%)
0
3
4
5
Time after telomere uncapping (h)
Time after telomere uncapping (h)
34°C
WT
mec3Δ
3' ssDNA (%)
0
4
5
Time after telomere uncapping (h)
Time after telomere uncapping (h)

Figure S3

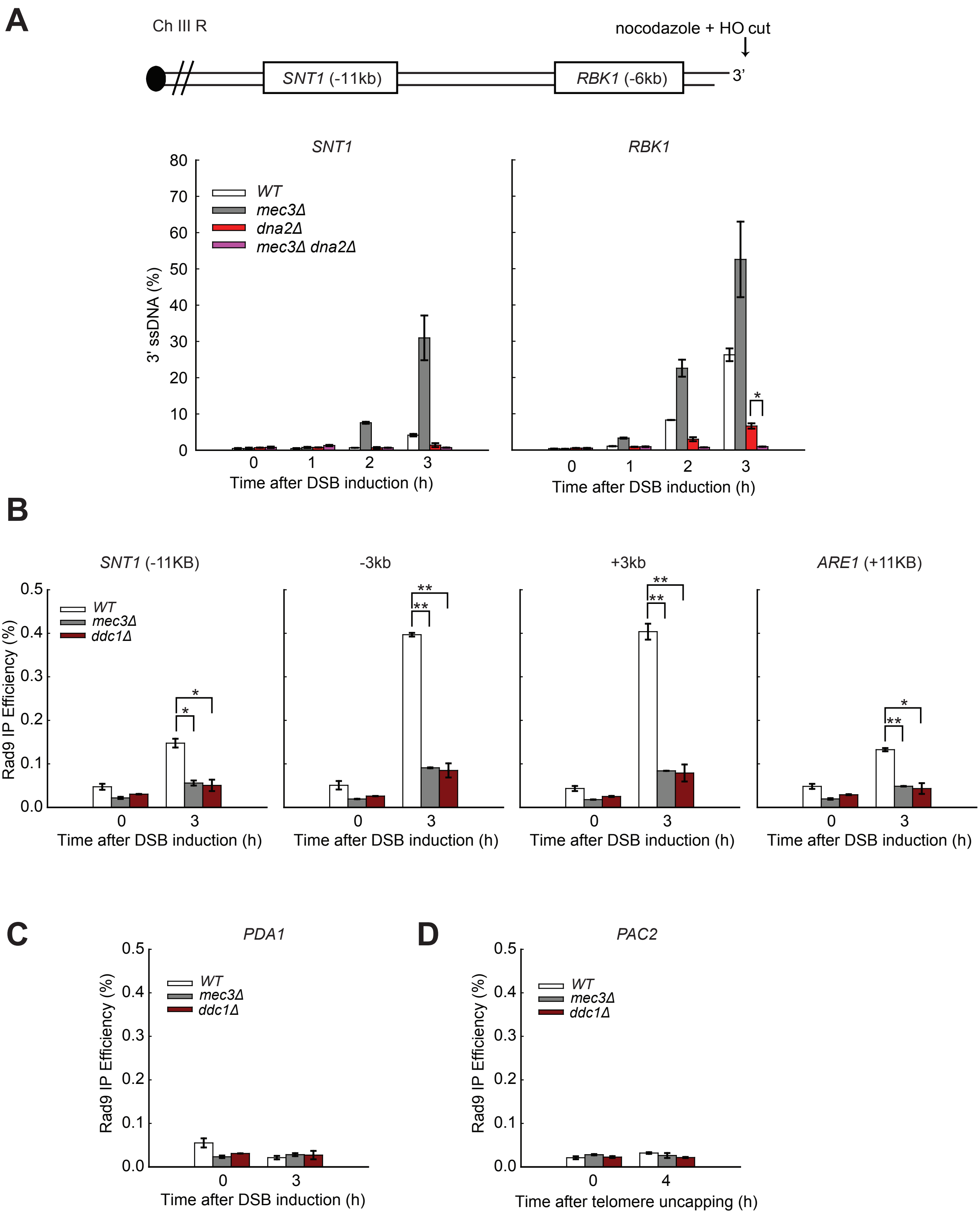

A
Ch III R
nocodazole + HO cut
SNT1 (-11kb)
RBK1 (-6kb)
3'
SNT1
RBK1
WT
mec3Δ
dna2Δ
mec3Δ dna2Δ
3' ssDNA (%)
0
10
20
30
40
50
60
70
80
Time after DSB induction (h)
B
SNT1 (-11KB)
-3kb
+3kb
ARE1 (+11KB)
Rad9 IP Efficiency (%)
WT
mec3Δ
ddc1Δ
0.0
0.1
0.2
0.3
0.4
0.5
Time after DSB induction (h)
C
PDA1
D
PAC2
Time after telomere uncapping (h)

Figure S4

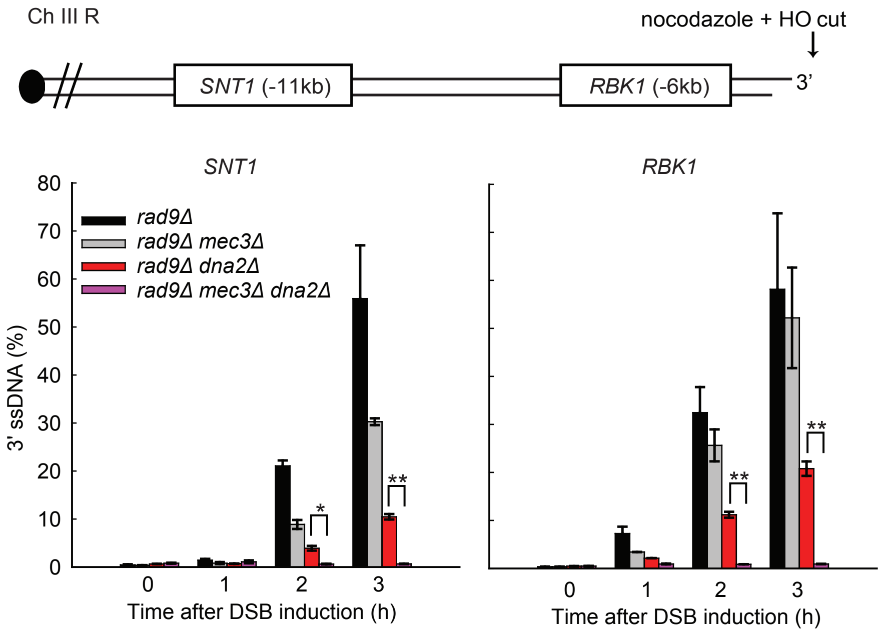

Ch III R
nocodazole + HO cut
3'
SNT1 (-11kb)
RBK1 (-6kb)
SNT1
RBK1
3' ssDNA (%)
0
10
20
30
40
50
60
70
80
rad9Δ
rad9Δ mec3Δ
rad9Δ dna2Δ
rad9Δ mec3Δ dna2Δ
*
**
0
1
2
3
Time after DSB induction (h)
Time after DSB induction (h)

Figure S5

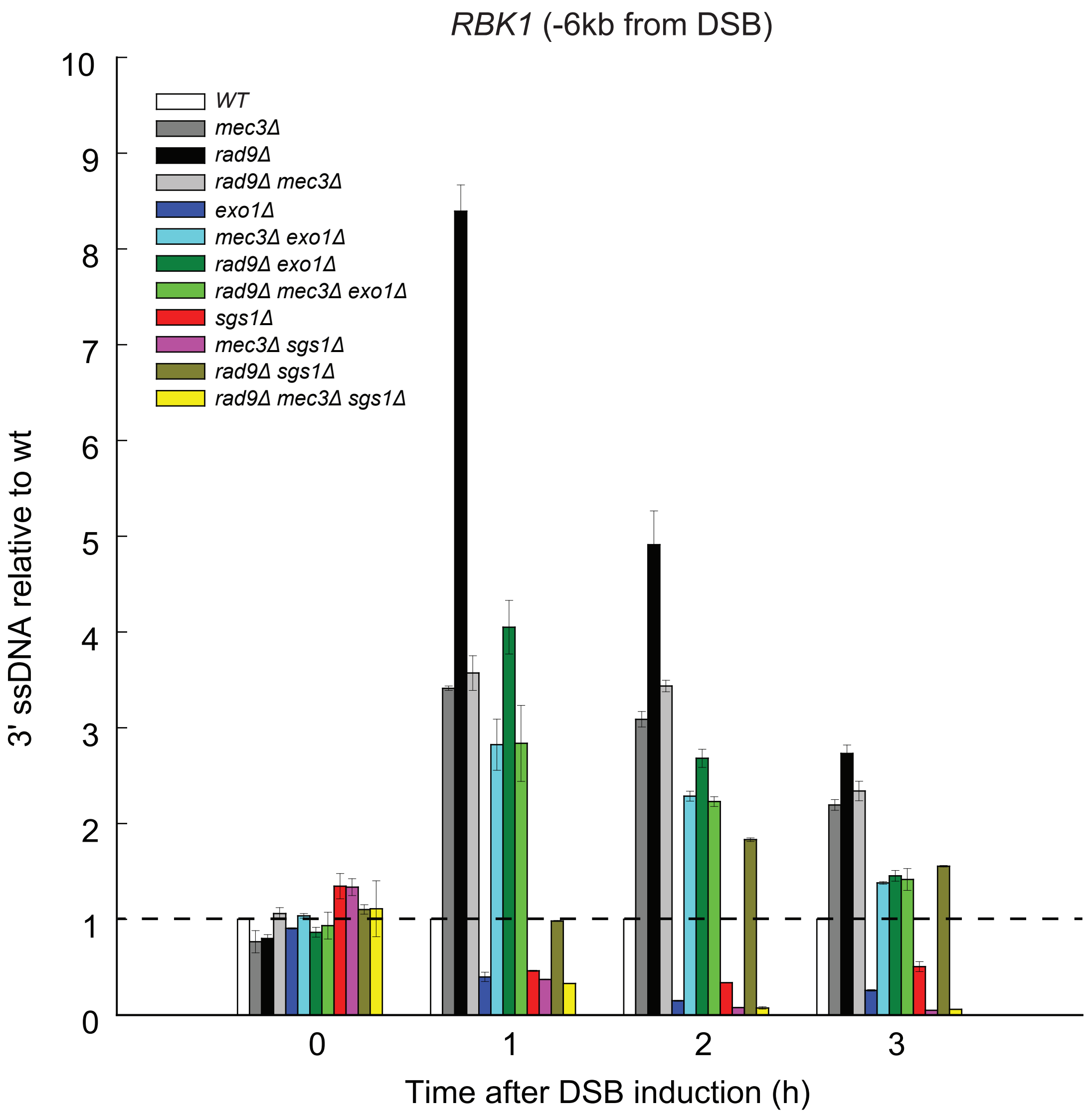
RBK1 (-6kb from DSB)
3' ssDNA relative to wt
0
1
2
3
4
5
6
7
8
9
10
WT
mec3Δ
rad9Δ
rad9Δ mec3Δ
exo1Δ
mec3Δ exo1Δ
rad9Δ exo1Δ
rad9Δ mec3Δ exo1Δ
sgs1Δ
mec3Δ sgs1Δ
rad9Δ sgs1Δ
rad9Δ mec3Δ sgs1Δ
0
1
2
3
Time after DSB induction (h)

Figure S6

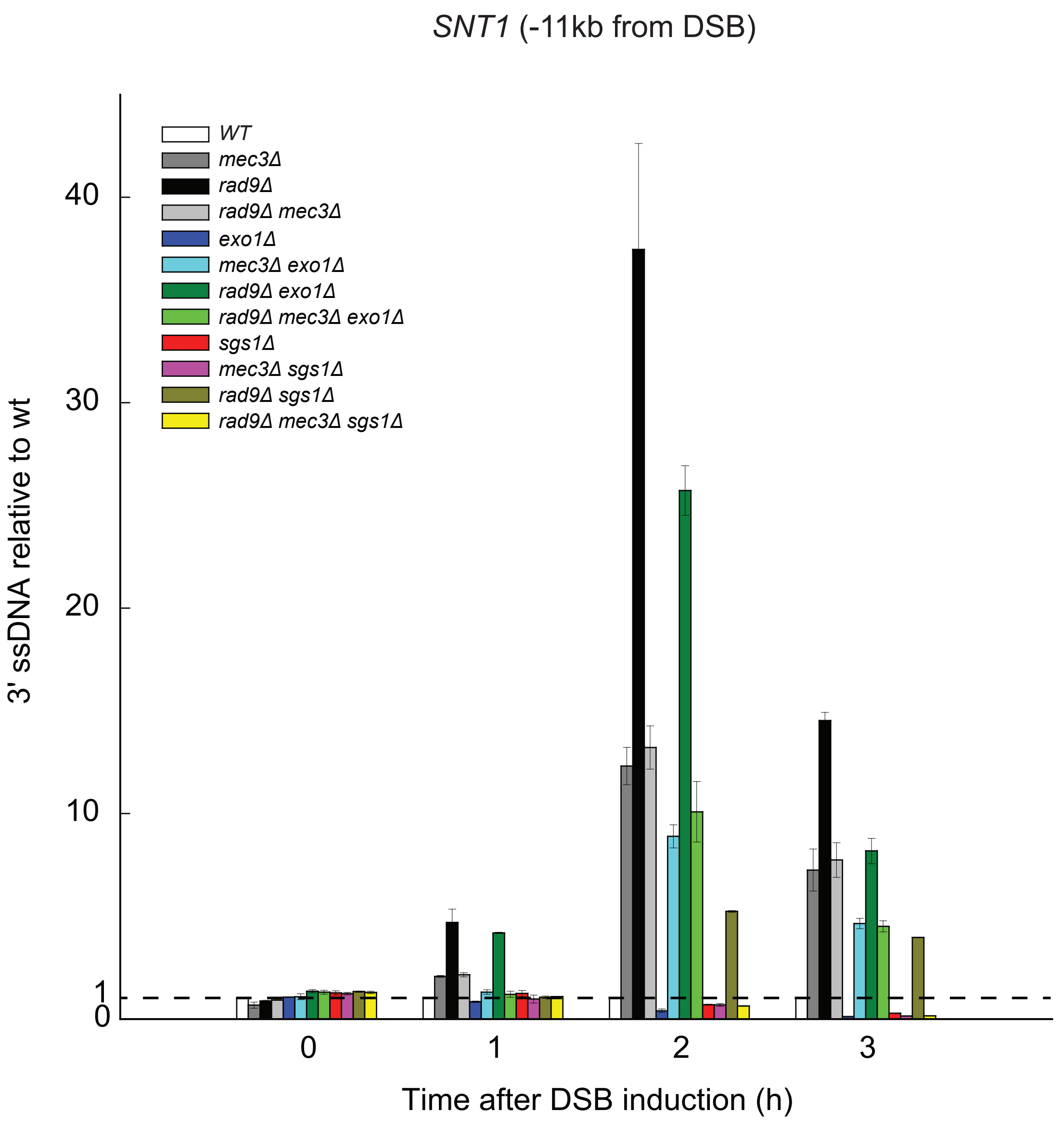

SNT1 (-11kb from DSB)
WT
mec3Δ
rad9Δ
rad9Δ mec3Δ
exo1Δ
mec3Δ exo1Δ
rad9Δ exo1Δ
rad9Δ mec3Δ exo1Δ
sgs1Δ
mec3Δ sgs1Δ
rad9Δ sgs1Δ
rad9Δ mec3Δ sgs1Δ
3' ssDNA relative to wt
40
30
20
10
1
0
0
1
2
3
Time after DSB induction (h)

Figure S7

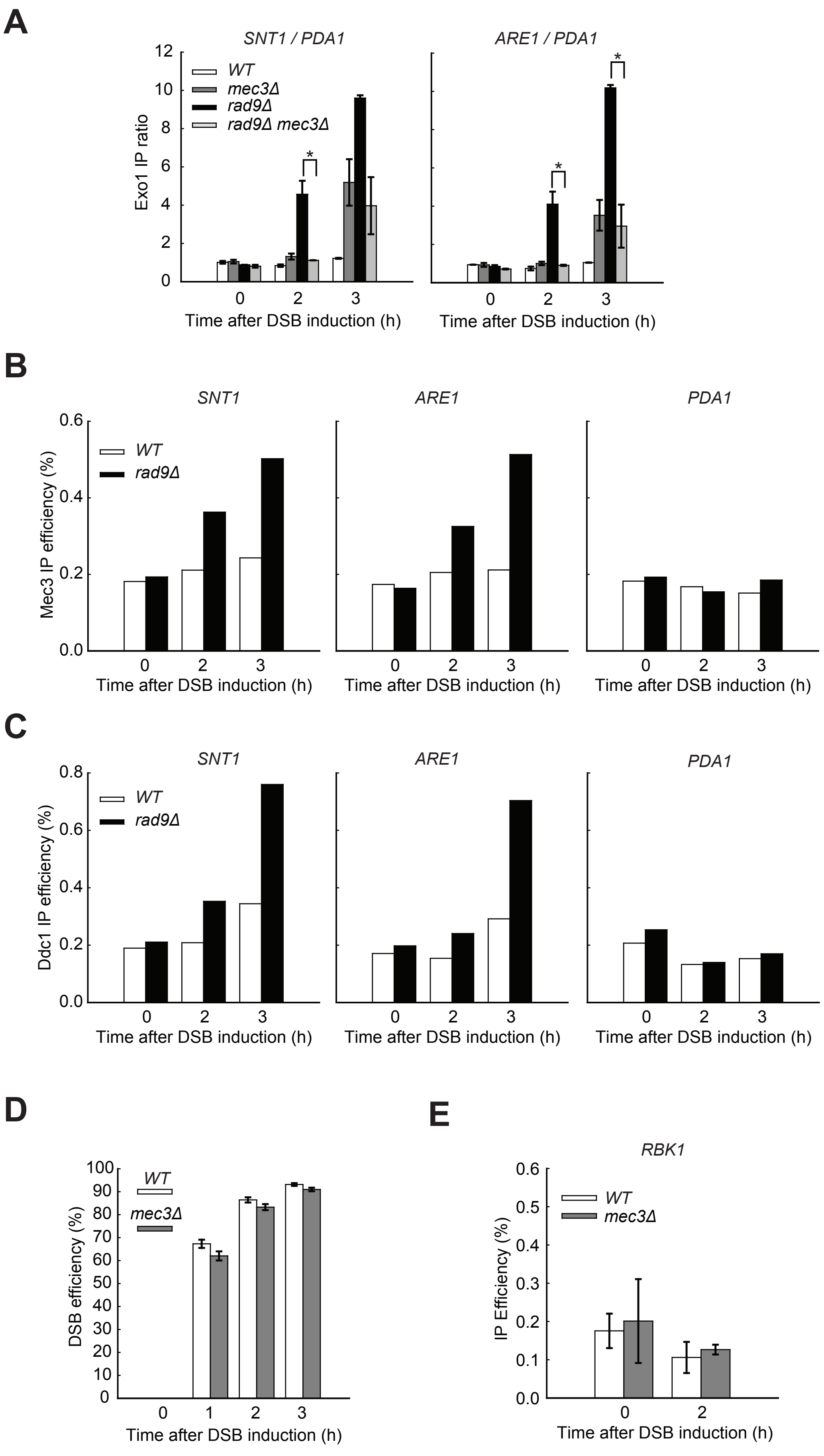
A
SNT1 / PDA1
ARE1 / PDA1
WT
mec3Δ
rad9Δ
rad9Δ mec3Δ
Exo1 IP ratio
0
2
4
6
8
10
12
*
Time after DSB induction (h)
B
SNT1
ARE1
PDA1
WT
rad9Δ
Mec3 IP efficiency (%)
0.0
0.2
0.4
0.6
Time after DSB induction (h)
C
SNT1
ARE1
PDA1
WT
rad9Δ
Ddc1 IP efficiency (%)
0.0
0.2
0.4
0.6
0.8
Time after DSB induction (h)
D
WT
mec3Δ
DSB efficiency (%)
0
10
20
30
40
50
60
70
80
90
100
0
1
2
3
Time after DSB induction (h)
E
RBK1
WT
mec3Δ
IP Efficiency (%)
0.0
0.1
0.2
0.3
0.4
0.5
0.6
0
2
Time after DSB induction (h)

Table S1

Strains	Genotype	Source
DLY7846/ 7847	*MATalpha hoΔ hml::ADE1 hmr::ADE1 ade1-100 leu2-3,112 trp1::hisG lys5 ura3-52 ade3::GAL::HO* (JKM179)	M.Muzi-Falconi Fig. 1
DLY7848/ 7849	*MATalpha hoΔ hml::ADE1 hmr::ADE1 ade1-100 leu2-3,112 trp1::hisG lys5 ura3-52 ade3::GAL::HO rad9Δ::TRP1* (YFL419)	M.Muzi-Falconi Fig. 3
DLY7982/ 7983	*MATalpha hoΔ hml::ADE1 hmr::ADE1 ade1-100 leu2-3,112 trp1::hisG lys5 ura3-52 ade3::GAL::HO mec3Δ::HphMX*	This work, Fig.1
DLY7986/ 7987	*MATalpha hoΔ hml::ADE1 hmr::ADE1 ade1-100 leu2-3,112 trp1::hisG lys5 ura3-52 ade3::GAL::HO rad9Δ::TRP1 mec3Δ::HphMX*	This work, Fig.3
DLY8299/ 8300	*MATalpha hoΔ hml::ADE1 hmr::ADE1 ade1-100 leu2-3,112 trp1::hisG lys5 ura3-52 ade3::GAL::HO exo1Δ::URA3*	This work, Fig.2
DLY8301/ 8302	*MATalpha hoΔ hml::ADE1 hmr::ADE1 ade1-100 leu2-3,112 trp1::hisG lys5 ura3-52 ade3::GAL::HO rad9Δ::TRP1 exo1Δ::URA3*	This work, Fig.5
DLY8339/ 8340	*MATalpha hoΔ hml::ADE1 hmr::ADE1 ade1-100 leu2-3,112 trp1::hisG lys5 ura3-52 ade3::GAL::HO mec3Δ::HphMX exo1Δ::URA3*	This work, Fig.2
DLY8341/ 8342	*MATalpha hoΔ hml::ADE1 hmr::ADE1 ade1-100 leu2-3,112 trp1::hisG lys5 ura3-52 ade3::GAL::HO rad9Δ::TRP1 mec3Δ::HphMX exo1Δ::URA3*	This work, Fig.5
DLY8304	*MATalpha hoΔ hml::ADE1 hmr::ADE1 ade1-100 leu2-3,112 trp1::hisG lys5 ura3-52 ade3::GAL::HO pif1Δ::KanMX*	This work, Fig.2
DLY8307	*MATalpha hoΔ hml::ADE1 hmr::ADE1 ade1-100 leu2-3,112 trp1::hisG lys5 ura3-52 ade3::GAL::HO rad9Δ::TRP1 pif1Δ::KanMX*	This work, Fig.5
DLY8335	*MATalpha hoΔ hml::ADE1 hmr::ADE1 ade1-100 leu2-3,112 trp1::hisG lys5 ura3-52 ade3::GAL::HO mec3Δ::HphMX pif1Δ::KanMX*	This work, Fig.2
DLY8337	*MATalpha hoΔ hml::ADE1 hmr::ADE1 ade1-100 leu2-3,112 trp1::hisG lys5 ura3-52 ade3::GAL::HO rad9Δ::TRP1 mec3Δ::HphMX pif1Δ::KanMX*	This work, Fig.5
DLY8393/ 8394	*MATalpha hoΔ hml::ADE1 hmr::ADE1 ade1-100 leu2-3,112 trp1::hisG lys5 ura3-52 ade3::GAL::HO pif1Δ::KanMX dna2Δ::NatMX*	This work, Fig.S3
DLY8396/ 8397	*MATalpha hoΔ hml::ADE1 hmr::ADE1 ade1-100 leu2-3,112 trp1::hisG lys5 ura3-52 ade3::GAL::HO rad9Δ::TRP1 pif1Δ::KanMX dna2Δ::NatMX*	This work, Fig.S4
DLY8399/ 8400	*MATalpha hoΔ hml::ADE1 hmr::ADE1 ade1-100 leu2-3,112 trp1::hisG lys5 ura3-52 ade3::GAL::HO mec3Δ::HphMX pif1Δ::KanMX dna2Δ::NatMX*	This work, Fig.S3
DLY8402/ 8403	*MATalpha hoΔ hml::ADE1 hmr::ADE1 ade1-100 leu2-3,112 trp1::hisG lys5 ura3-52 ade3::GAL::HO rad9Δ::TRP1 mec3Δ::HphMX pif1Δ::KanMX dna2Δ::NatMX*	This work, Fig.S4
DLY8578/ 8579	*MATalpha hoΔ hml::ADE1 hmr::ADE1 ade1-100 leu2-3,112 trp1::hisG lys5 ura3-52 ade3::GAL::HO sgs1Δ::KanMX exo1Δ::URA3*	This work, Fig.2
DLY8580/ 8581	*MATalpha hoΔ hml::ADE1 hmr::ADE1 ade1-100 leu2-3,112 trp1::hisG lys5 ura3-52 ade3::GAL::HO rad9Δ::TRP1 sgs1Δ::KanMX exo1Δ::URA3*	This work, Fig.5
DLY8582/ 8583	*MATalpha hoΔ hml::ADE1 hmr::ADE1 ade1-100 leu2-3,112 trp1::hisG lys5 ura3-52 ade3::GAL::HO mec3Δ::HphMX sgs1Δ::KanMX exo1Δ::URA3*	This work, Fig.2
DLY8584/ 8585	*MATalpha hoΔ hml::ADE1 hmr::ADE1 ade1-100 leu2-3,112 trp1::hisG lys5 ura3-52 ade3::GAL::HO rad9Δ::TRP1 mec3Δ::HphMX sgs1Δ::KanMX exo1Δ::URA3*	This work, Fig.5
DLY8329/ 8330	*MATalpha hoΔ hml::ADE1 hmr::ADE1 ade1-100 leu2-3,112 trp1::hisG lys5 ura3-52 ade3::GAL::HO ddc1Δ::HphMX*	This work, Fig.1
DLY8332/ 8333	*MATalpha hoΔ hml::ADE1 hmr::ADE1 ade1-100 leu2-3,112 trp1::hisG lys5 ura3-52 ade3::GAL::HO rad9Δ::TRP1 ddc1Δ::HphMX*	This work, Fig.3
DLY9504/ 9505	*MATalpha hoΔ hml::ADE1 hmr::ADE1 ade1-100 leu2-3,112 trp1::hisG lys5 ura3-52 ade3::GAL::HO Rad9-HA-KanMX*	This work, Fig.3
DLY9506/ 9507	*MATalpha hoΔ hml::ADE1 hmr::ADE1 ade1-100 leu2-3,112 trp1::hisG lys5 ura3-52 ade3::GAL::HO mec3Δ::HphMX Rad9-HA-KanMX*	This work, Fig.3
DLY9509/ 9511	*MATalpha hoΔ hml::ADE1 hmr::ADE1 ade1-100 leu2-3,112 trp1::hisG lys5 ura3-52 ade3::GAL::HO ddc1Δ::HphMX Rad9-HA-KanMX*	This work, Fig.3
DLY7942/ 7943	*MATalpha hoΔ hml::ADE1 hmr::ADE1 ade1-100 leu2-3,112 trp1::hisG lys5 ura3-52 ade3::GAL::HO Dna2-Myc-KanMX*	This work, Fig.6
DLY7945/ 7946	*MATalpha hoΔ hml::ADE1 hmr::ADE1 ade1-100 leu2-3,112 trp1::hisG lys5 ura3-52 ade3::GAL::HO rad9Δ::TRP1 Dna2-Myc-KanMX*	This work, Fig.6
DLY7988/ 7989	*MATalpha hoΔ hml::ADE1 hmr::ADE1 ade1-100 leu2-3,112 trp1::hisG lys5 ura3-52 ade3::GAL::HO mec3Δ::HphMX Dna2-Myc-KanMX*	This work, Fig.6
DLY7991/ 7992	*MATalpha hoΔ hml::ADE1 hmr::ADE1 ade1-100 leu2-3,112 trp1::hisG lys5 ura3-52 ade3::GAL::HO rad9Δ::TRP1 mec3Δ::HphMX Dna2-Myc-KanMX*	This work, Fig.6

Table S2

Strains	Genotype	Source
DLY8060/ 8061	*MATalpha hoΔ hml::ADE1 hmr::ADE1 ade1-100 leu2-3,112 trp1::hisG lys5 ura3-52 ade3::GAL::HO Exo1-Myc-KanMX*	This work, Fig.6
DLY8075/ 8076	*MATalpha hoΔ hml::ADE1 hmr::ADE1 ade1-100 leu2-3,112 trp1::hisG lys5 ura3-52 ade3::GAL::HO rad9Δ::TRP1 Exo1-Myc-KanMX*	This work, Fig.6
DLY8187/ 8188	*MATalpha hoΔ hml::ADE1 hmr::ADE1 ade1-100 leu2-3,112 trp1::hisG lys5 ura3-52 ade3::GAL::HO mec3Δ::HphMX Exo1-Myc-KanMX*	This work, Fig.6
DLY8190/ 8191	*MATalpha hoΔ hml::ADE1 hmr::ADE1 ade1-100 leu2-3,112 trp1::hisG lys5 ura3-52 ade3::GAL::HO rad9Δ::TRP1 mec3Δ::HphMX Exo1-Myc-KanMX*	This work, Fig.6
DLY8051	*MATalpha hoΔ hml::ADE1 hmr::ADE1 ade1-100 leu2-3,112 trp1::hisG lys5 ura3-52 ade3::GAL::HO Mec3-Myc-KanMX*	This work, Fig.6
DLY8066	*MATalpha hoΔ hml::ADE1 hmr::ADE1 ade1-100 leu2-3,112 trp1::hisG lys5 ura3-52 ade3::GAL::HO rad9Δ::TRP1 Mec3-Myc-KanMX*	This work, Fig.6
DLY8054	*MATalpha hoΔ hml::ADE1 hmr::ADE1 ade1-100 leu2-3,112 trp1::hisG lys5 ura3-52 ade3::GAL::HO Ddc1-Myc-KanMX*	This work, Fig.6
DLY8069	*MATalpha hoΔ hml::ADE1 hmr::ADE1 ade1-100 leu2-3,112 trp1::hisG lys5 ura3-52 ade3::GAL::HO rad9Δ::TRP1 Ddc1-Myc-KanMX*	This work, Fig.6
DLY9513/ 9514	*MAT a ade2-1 trp1-1 can1-100 leu2-3,112 his3-11,15 ura3* GAL+ psi+ ssd1-d2 RAD5 *cdc13-1 cdc15-2 bar1 Rad9-HA-KanMX*	This work, Fig.3
DLY9515/ 9516	*MAT a ade2-1 trp1-1 can1-100 leu2-3,112 his3-11,15 ura3* GAL+ psi+ ssd1-d2 RAD5 *cdc13-1 cdc15-2 bar1 Rad9-HA-KanMX mec3Δ::TRP1*	This work, Fig.3
DLY9519/ 9520	*MAT a ade2-1 trp1-1 can1-100 leu2-3,112 his3-11,15 ura3* GAL+ psi+ ssd1-d2 RAD5 *cdc13-1 cdc15-2 bar1 Rad9-HA-KanMX ddc1Δ::HphMX*	This work, Fig.3
DLY10009	*MATalpha hoΔ hml::ADE1 hmr::ADE1 ade1-100 leu2-3,112 trp1::hisG lys5 ura3-52 ade3::GAL::HO sgs1Δ::KanMX*	This work, Fig.2
DLY10013	*MATalpha hoΔ hml::ADE1 hmr::ADE1 ade1-100 leu2-3,112 trp1::hisG lys5 ura3-52 ade3::GAL::HO mec3Δ::HphMX sgs1Δ::KanMX*	This work, Fig.2
DLY8577	*MATalpha hoΔ hml::ADE1 hmr::ADE1 ade1-100 leu2-3,112 trp1::hisG lys5 ura3-52 ade3::GAL::HO rad9Δ::TRP1 sgs1Δ::KanMX*	This work, Fig.5
DLY8069	*MATalpha hoΔ hml::ADE1 hmr::ADE1 ade1-100 leu2-3,112 trp1::hisG lys5 ura3-52 ade3::GAL::HO rad9Δ::TRP1 mec3Δ::HphMX sgs1Δ::KanMX*	This work, Fig.5
DLY10101	*Mata-inc ura3::HOcs(V) LYS2 ade3::GALHO leu2-3,112 his3-11,15 trp1-1 ade2-1 can1-100*	M.Kupiec, Fig.7
DLY10108/ 10109	*Mata-inc ura3::HOcs(V) LYS2 ade3::GALHO leu2-3,112 his3-11,15 trp1-1 ade2-1 can1-100 mec3Δ::HphMX*	This work, Fig.7
DLY10242/ 10243	*Mata-inc ura3::HOcs(V) LYS2 ade3::GALHO leu2-3,112 his3-11,15 trp1-1 ade2-1 can1-100 rad9Δ::KanMX*	This work, Fig.7
DLY10244/ 10245	*Mata-inc ura3::HOcs(V) LYS2 ade3::GALHO leu2-3,112 his3-11,15 trp1-1 ade2-1 can1-100 rad9Δ::KanMX mec3Δ::HphMX*	This work, Fig.7
DLY10108/ 10109	*Mata-inc ura3::HOcs(V) LYS2 ade3::GALHO leu2-3,112 his3-11,15 trp1-1 ade2-1 can1-100 Rad9-HA-KanMX*	This work, Fig.7
DLY10242/ 10243	*Mata-inc ura3::HOcs(V) LYS2 ade3::GALHO leu2-3,112 his3-11,15 trp1-1 ade2-1 can1-100 mec3Δ::HphMX Rad9-HA-KanMX*	This work, Fig.7

Table S3

Loci	Primer	Sequence (5'-3')	Type
ARE1 3'	M2646	TTTGATCCTCGCCCCACGCTCCTGGAC	Probe
	M2651	TCGTCTGGAAGGGCACGTTGATG	Reverse
	M239	AACCAGCGCAGCGGCATGTGT	Tag
	M2679	AACCAGCGCAGCGGCATGTGTGTCGTTTTTTG	Tagging
YCR043C 3'	M2649	CTTGCTGTCCAAGCCAACATTCCCGCCA	Probe
	M2657	GAGATAGGAGGAGAAAAAACAGCAGAAGTATACTGA	Reverse
	M235	TGCCCTCGCATCGCTCTCACA	Tag
	M2674	TGCCCTCGCATCGCTCTCACAGTGAAAGACCG	Tagging
SNT1 3'	M2648	TCCCTTTACCGGCCCCTCGCACTAGTCCA	Probe
	M2654	TCAGCATGCTATTTCTCAAGGCACTCCTA	Reverse
	M97	GATCTCGAGCTCGATATCGGATCCATT	Tag
	M2660	GATCTCGAGCTCGATATCGGATCCATTCGAAAAATTGA	Tagging
RBK1 3'	M2647	CATGAGGCTAACAAGGCGCAACCGAACACA	Probe
	M2652	AATCCAGATGAAGCCAACCCCATACC	Reverse
	M234	AAGGAGCGCAGCGCCTGTACCA	Tag
	M2659	AAGGAGCGCAGCGCCTGTACCAAAGGCAGCT	Tagging
ARE1 5'	M2646	TTTGATCCTCGCCCCACGCTCCTGGAC	Probe
	M2650	TCCACCGAAAGGATGCTAGCAAGTATGT	Reverse
	M418	ATGCTCGCAGAGCCCCTGGATCT	Tag
	M2658	ATGCTCGCAGAGCCCCTGGATCTGGAAGGGCA	Tagging
SNT1 5'	M2648	TCCCTTTACCGGCCCCTCGCACTAGTCCA	Probe
	M2655	CAAAGCTGCCAACGGATCATTCG	Reverse
	M418	ATGCTCGCAGAGCCCCTGGATCT	Tag
	M2678	ATGCTCGCAGAGCCCCTGGATCTCAAGGCACTC	Tagging
YCR043C 5'	M2649	CTTGCTGTCCAAGCCAACATTCCCGCCA	Probe
	M2656	CCGTTATGATCAAATCACGACAGTGAAAGAC	Reverse
	M235	TGCCCTCGCATCGCTCTCACA	Tag
	M2662	TGCCCTCGCATCGCTCTCACAGCAGAAGTATACT	Tagging
RBK1 5'	M2647	CATGAGGCTAACAAGGCGCAACCGAACACA	Probe
	M2653	CCGATCCAAAGGCAGCTCCAA	Reverse
	M234	AAGGAGCGCAGCGCCTGTACCA	Tag
	M2675	AAGGAGCGCAGCGCCTGTACCAGATGAAGCC	Tagging

Table S4

Loci	Primer	Sequence (5'-3')	Type
GEA2 3'	M3638<br>M3637<br>M97<br>M3659	TCCTCCCCCCAGCAAAGCGGCCACT<br>CGACAGCGATGCGGAAATACTCTAAATTTA<br>GATCTCGAGCTCGATATCGGATCCATT<br>GATCTCGAGCTCGATATCGGATCCATTGTTGAATGTGT	Probe<br>Reverse<br>Tag<br>Tagging
RIP1 3'	M3642<br>M3640<br>M520<br>M3668	AAGGGAGCCTCCGCGTCGCCGCA<br>CGTTTTGCGCCATTTATCGGTCTTG<br>TGCCCTCGCATCGCTCTCGAA<br>TGCCCTCGCATCGCTCTCGAACCAACTATAGT	Probe<br>Reverse<br>Tag<br>Tagging
EAF5 3'	M3632<br>M3631<br>M233<br>M3664	ACCGGTGCATCGCAACTGCAACCACATG<br>CAGCAGATCCACTTTTGCCAGCATT<br>ATGCCCGCACCGCCTCATTG<br>ATGCCCGCACCGCCTCATTGAAGAACGGTAT	Probe<br>Reverse<br>Tag<br>Tagging
NPP2 3'	M3635<br>M3633<br>M239<br>M3654	TTCCCGGCCGACGCAATGAGCGC<br>CAACGCGAATGACGGTGAGCAT<br>AACCAGCGCAGCGGCATGTGT<br>AACCAGCGCAGCGGCATGTGTCTCGAGGGT	Probe<br>Reverse<br>Tag<br>Tagging
Primers flanking *MAT* HO site	M3701<br>M3704	AAAATGCAGCACGGAATATG<br>TCCGTCACCACGTACTTCAG	Forward<br>Reverse
Primers flanking *URA3* HO site	M3707<br>M3708	GCATTAGGTCCCAAAATTTGTT<br>TTGGCGGATAATGCCTTTAG	Forward<br>Reverse
